# Promoting Apoptosis, a Promising Way to Treat Breast Cancer With Natural Products: A Comprehensive Review

**DOI:** 10.3389/fphar.2021.801662

**Published:** 2022-01-28

**Authors:** Lie Yuan, Yongqing Cai, Liang Zhang, Sijia Liu, Pan Li, Xiaoli Li

**Affiliations:** ^1^ Department of Pharmacology, College of Pharmacy, Chongqing Medical University, Chongqing, China; ^2^ Chongqing Key Laboratory of Drug Metabolism, Chongqing, China; ^3^ Key Laboratory for Biochemistry and Molecular Pharmacology of Chongqing, Chongqing, China; ^4^ Department of Pharmacy, Daping Hospital, Army Medical University, Chongqing, China; ^5^ Department of Pharmacy, Fengdu County Hospital of Traditional Chinese Medicine, Chongqing, China

**Keywords:** breast cancer, apoptosis, natural products, monomer, mechanism

## Abstract

Breast cancer is one of the top-ranked malignant carcinomas associated with morbidity and mortality in women worldwide. Chemotherapy is one of the main approaches to breast cancer treatment. Breast cancer initially responds to traditional first- and second-line drugs (aromatase inhibitor, tamoxifen, and carboplatin), but eventually acquires resistance, and certain patients relapse within 5 years. Chemotherapeutic drugs also have obvious toxic effects. In recent years, natural products have been widely used in breast cancer research because of their low side effects, low toxicity, and good efficacy based on their multitarget therapy. Apoptosis, a programmed cell death, occurs as a normal and controlled process that promotes cell growth and death. Inducing apoptosis is an important strategy to control excessive breast cancer cell proliferation. Accumulating evidence has revealed that natural products become increasingly important in breast cancer treatment by suppressing cell apoptosis. In this study, we reviewed current studies on natural product–induced breast cancer cell apoptosis and summarized the proapoptosis mechanisms including mitochondrial, FasL/Fas, PI3K/AKT, reactive oxygen species, and mitogen-activated protein kinase–mediated pathway. We hope that our review can provide direction in the search for candidate drugs derived from natural products to treat breast cancer by promoting cell apoptosis.

## Introduction

Breast cancer (BC) is one of the most common malignancies occurring in women, with its morbidity increasing annually, thereby threatening human health (Georgalas et al., 2015). BC is characterized by high incidence, high mortality, high heterogeneity, high recurrence rate, and poor prognosis (Qian et al., 2019), which have collectively led to its morbidity and mortality surpassing that of lung cancer in women (Sung et al., 2021).

BC can mainly be divided into three subtypes according to histopathological features: estrogen receptor–positive (ER^+^), HER2-positive (HER2^+^), and triple-negative (TN). ER^+^ BC can be treated with tamoxifen and aromatase inhibitors. HER2^+^ BC can be treated with monoclonal antibodies against HER2, such as trastuzumab. TNBC does not express specific molecules; therefore, standard cytotoxic chemotherapy (doxorubicin, docetaxel, 5-fluorouracil, platinum drugs, and other agents in different combinations) remains the standard of care for patients with TNBC (Nøhr-Nielsen et al., 2020; Su et al., 2020; Pal and Rakshit, 2021; Shimanuki et al., 2021). However, regardless of the treatment strategy, the potential side effects, including lymphedema, weight reduction, pain, and chemotherapy-induced peripheral neuropathy, are significant (Binkley et al., 2012). Furthermore, BC initially responds to traditional chemotherapy. However, it eventually acquires resistance and metastasis in the advanced stage. Moreover, certain patients relapse within 5 years. Therefore, the discovery of additional or substituted drugs to treat BC is urgently required.

In the last 2 decades, natural products, such as alkaloids, flavonoids, terpenoids, and phenylpropanoids, have been widely used in preclinical studies of BC because of their rich natural resources, low toxicity and good efficacy (Ateba et al., 2018; Hematpoor et al., 2018; Kushwaha et al., 2020; Sindhu et al., 2021). They can target various apoptosis-related signaling pathways (PI3K/AKT, mitogen-activated protein kinase [MAPK], and p53) to induce apoptosis in BC cells (Khojasteh Poor et al., 2021; Li et al., 2021). Therefore, natural products are an important resource in the search for candidate drugs to treat BC. However, there is no systematic analysis or review of natural products for treating BC by promoting apoptosis. Herein, the purpose of our review is to summarize the latest research on natural products, including monomers and extracts, on anti-BC treatment through different molecular mechanisms to induce cell apoptosis. We hope that this article will provide direction for follow-up studies of natural products for BC treatment.

## Apoptosis and BC

Cell death is indispensable in the growth, development, senescence, and death of an organism (Liu et al., 2018a). It can occur through numerous regulatory mechanisms, including apoptosis, necrosis, necroptosis, pyroptosis, and ferroptosis (Zhang et al., 2021b). Apoptosis and autophagy are known as type I and type II programmed cell death, respectively. Autophagy is a double-edged sword in tumor cells. It can degrade and recycle cellular components in an orderly manner to maintain homeostasis and promote the survival of tumor cells. However, excessive autophagy can lead to autophagic cell death (Yu et al., 2008). Unlike autophagy, apoptosis has been identified as a highly regulated and controlled process that promotes tumor cell death.

Apoptosis is a form of cellular suicide triggered by extracellular (extrinsic apoptosis) or intracellular (intrinsic apoptosis) signals (Suraweera et al., 2020). Its biochemical features include cell contraction, nuclear fragmentation, chromatin aggregation, DNA fragmentation, mRNA decay, and the formation of apoptotic bodies (Roos and Kaina, 2013). Excessive apoptosis leads to atrophy, whereas inadequate apoptosis is associated with uncontrolled cell proliferation, as observed in tumors. In normal breast cells, there is a balance between cell proliferation and apoptosis, antiapoptosis and proapoptosis to maintain the cell homeostasis (Parton et al., 2001). Once the balance is disrupted, activated antiapoptotic signal pathway or proapoptosis pathway deficiency can lead to uncontrolled cell proliferation, therapeutic resistance, and cancer cell recurrence (Mohammad et al., 2015). In BC cells, multiple factors, including growth factor, DNA damage, reactive oxygen species (ROS), and UV radiation, can promote uncontrolled cell growth through mitochondrial, FasL/Fas–, PI3K/AKT–, ROS-, nuclear factor κB (NF-κB)–, and MAPK–mediated pathways, to break the balance of proapoptotic and antiapoptotic effects. Therefore, targeting apoptotic pathways is an efficient strategy for identifying candidate drugs derived from natural products to treat BC (Rajabi et al., 2021).

## Proapoptotic Effects of Monomers From Natural Products on BC

An increasing number of studies have comprehensively demonstrated the proapoptotic effects of natural products on BC. In this study, we reviewed the effect and mechanism of monomers derived from natural products on apoptosis in BC (Table 1).

**TABLE 1:** Proapoptotic effects of monomers from natural products on breast cancer cells.

Monomers	Chemical structure	Detail mechanisms		Cell model	Refs
**Mitochondrial pathway–mediated apoptosis**					
Ganoderic acid DM (50 µM)	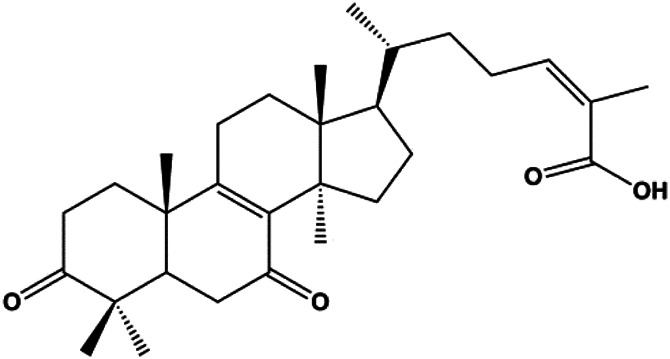	Cleaved PARP, γ-H2AX	CDK2, CDK6, p-Rb, CytoC	MCF-7	Wu et al. (2012)
18β-Glycyrrhetinic acid (100 µM)	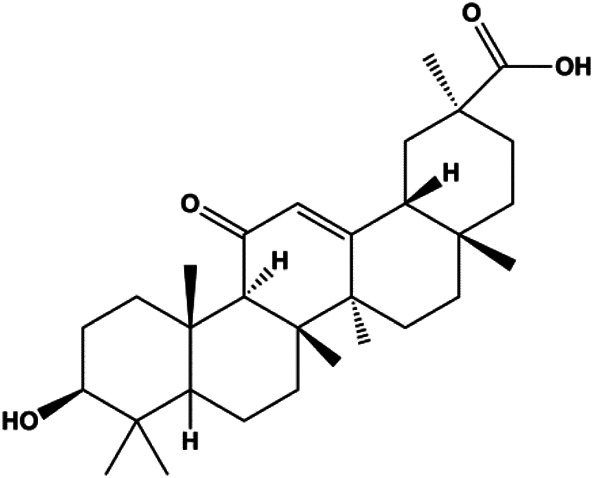	CytoC, activated caspase-9, Bax	Bcl-2	MCF-7	Sharma et al. (2012)
Apigenin (10, 20, 40, 80 μM)	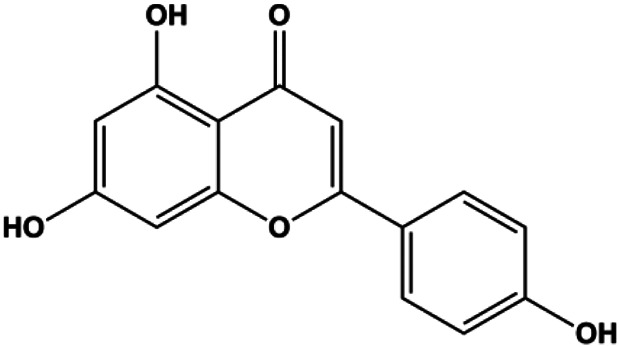	p53, p21, Bax, p-Cdc2, cleaved caspase-3, cleaved PARP	Bcl-2, cyclin B1, Cdc2	T47D	Liu et al. (2012)
Stevioside (10 µM)	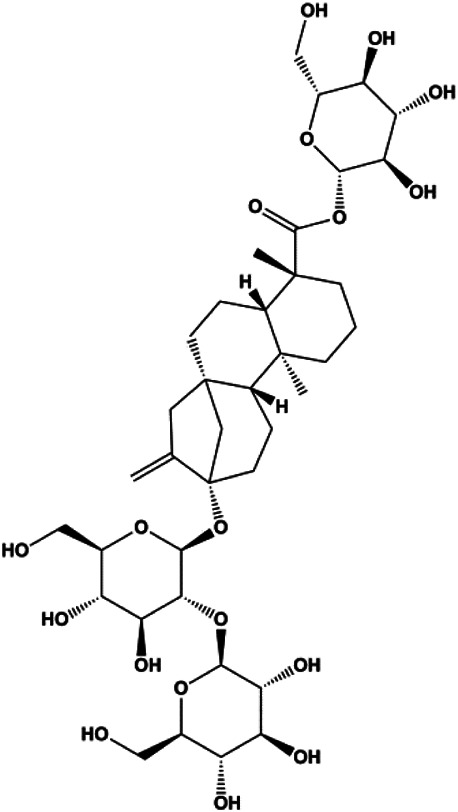	Bax, Caspase-9	Bcl-2	MCF7	Paul et al. (2012)
Amentoflavone (250 µM)	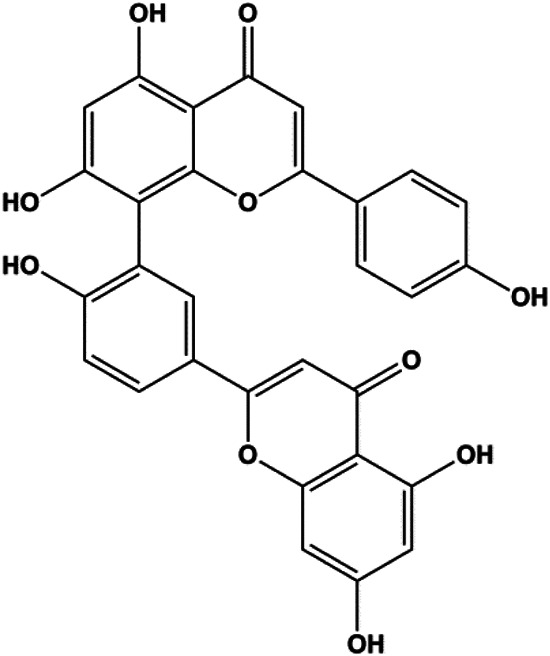	Bax, p53	Bid	MCF-7	Pei et al. (2012)
Calycosin (25, 50, 100 µM)	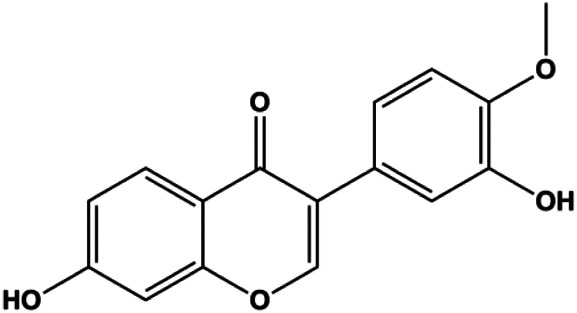	Bax	Bcl-2	MCF-7	Tian et al. (2013)
Thymoquinon (with 12.30 ± 0.62 μM in MDA-MB-468 with 18.06 ± 0.71 μM in T-47D)	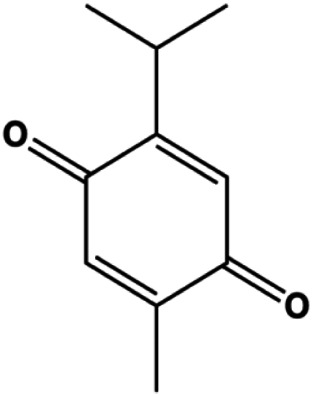	p27, Bax, CytoC, pro–caspase-3, cleaved PARP	Cyclin D1, cyclin E, Bcl-2, BclXL, survivin, p-PDK1, p-PTEN, p-Akt, p-GSK-3β, p-Bad, 4E-BP1, eIF4E, p-S6R, p-p70S6K	MDA-MB-468 T-47D	Rajput et al. (2013)
Embelin (40, 80 µg/mL)	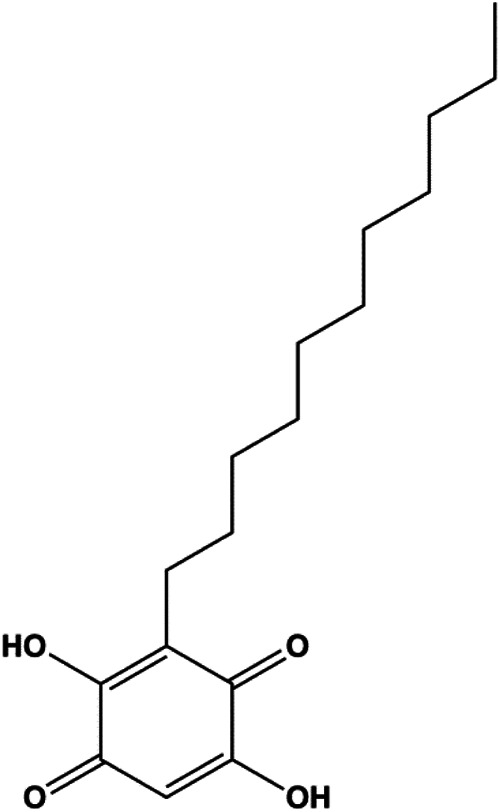	Bax, CytoC, caspase-3, caspase-9	Bcl-2	MCF-7	Li et al. (2013)
l-Carvone (0.3–2.4 mM)	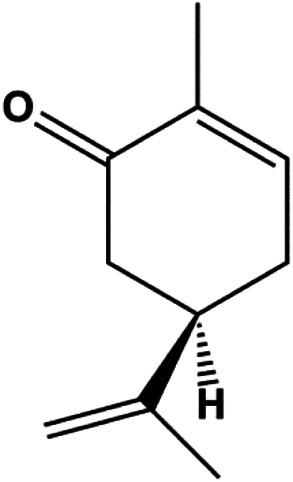	p53, Bad, cleaved caspase-3, cleaved PARP	MCF-7	MDA-MB-231	Patel and Thakkar, (2014)
Oleuropein (100, 200 μM)	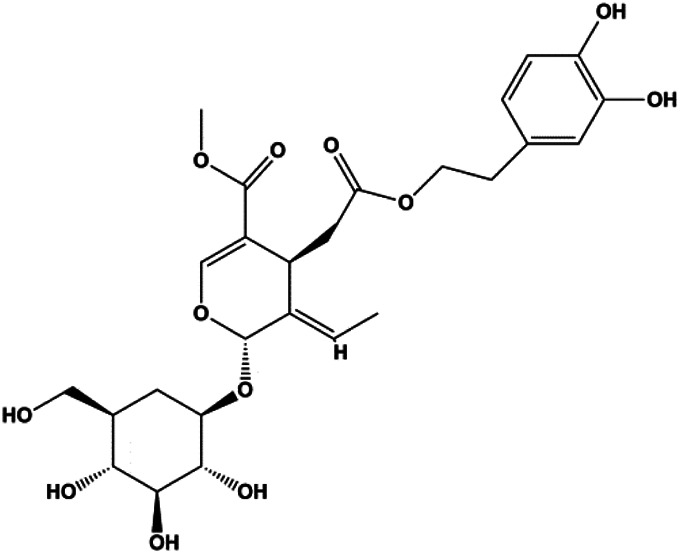	p53, Bax	Bcl-2	MCF-7	Hassan et al. (2014)
Ursolic acid (20, 40, 80 μmol/L)	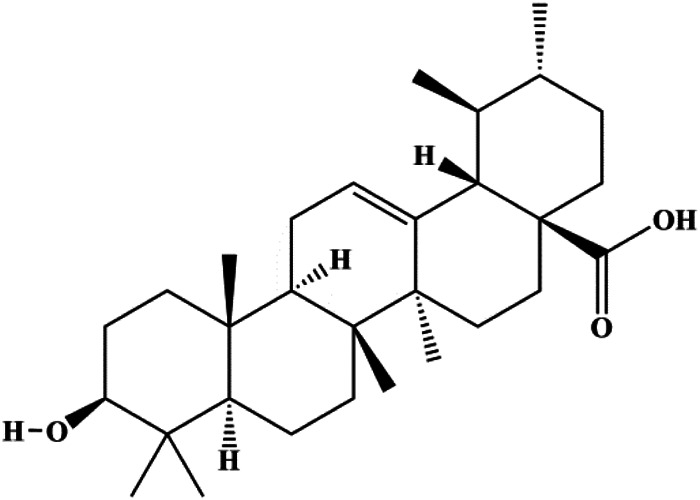	p16, p27, activated caspase-3, activated caspase-9		MDA-MB-231	Ma and Sun, (2014)
3β, 6β, 16β-trihydroxylup-20 (29)-ene (TTHL) (0.5, 1.36, 3.70 μg/mL)	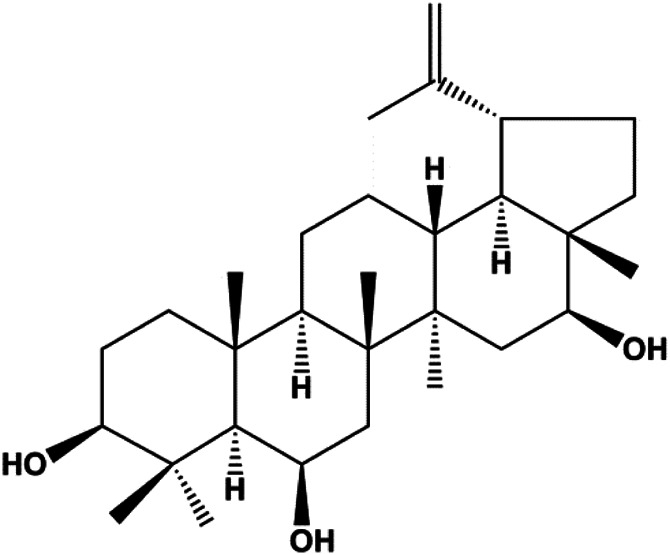	Cleaved caspase-9, ROS		MCF-7	Viau et al. (2014)
Dioscin (1, 2, 4, 6,8 μM)	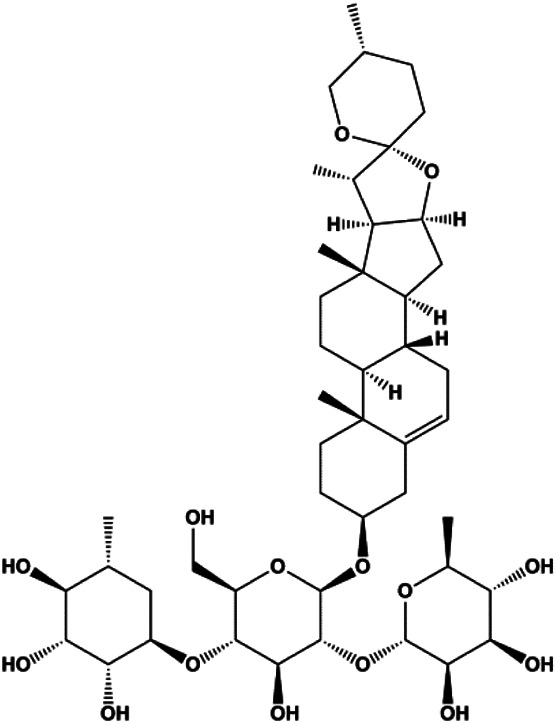	Cleaved caspase-3, cleaved PARP	Bcl-2, cIAP-1, Mcl-1	MDA-MB-231	Kim et al. (2014)
Emodin (10, 40 µM)	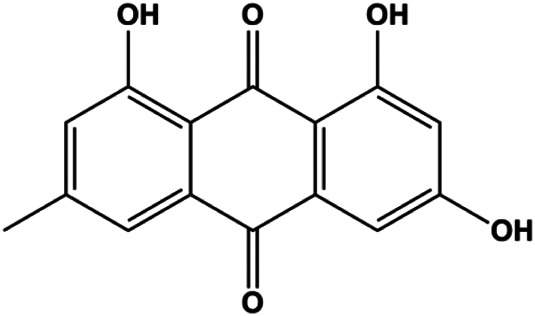	Cleaved caspase-3, PARP, p53, Bax	Bcl-2	Bcap-37 ZR-75-30	Zu et al. (2015)
Gaillardin (1, 10, 100 mM)	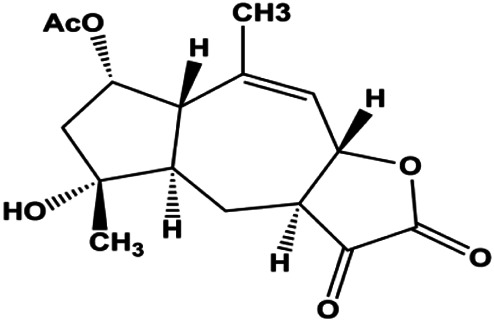	Bax, p53	Bcl-2	MCF-7 MDA-MB-468	Fallahian et al. (2015)
Sesamin (1, 10, 50 μM)	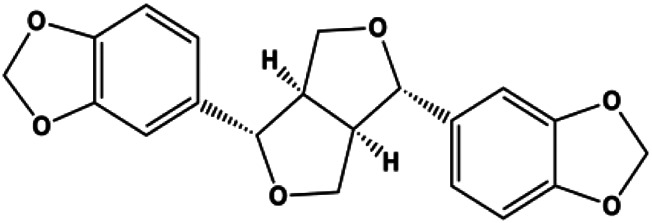	Bax, caspase-3, p53		MCF-7	Siao et al. (2015)
20(S)-protopanaxadiol (0–60 ml)	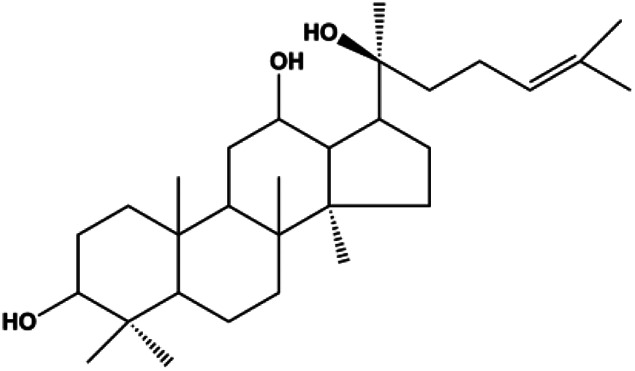	Bax, cleaved PARP, p53	Bcl-2, CytoC, cyclin D1, CDK4	MCF-7	Zhang et al. (2018a)
4-Methylthiobutyl isothiocyanate (2.5, 5, 10, 20 μM)	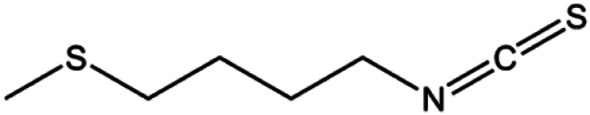	Cleaved caspase-9, cleaved caspase-3	PARP	MDA-MB-231 MCF-7	Li et al. (2015)
Paratocarpin E (10, 20, 30, 40 μM)	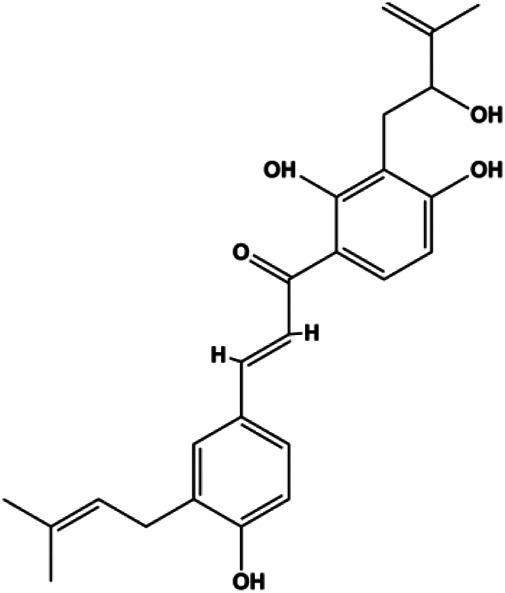	Cleaved caspase-8, cleaved caspase-9, Bax, CytoC	Bcl-2	MCF-7	Gao et al. (2016)
Arecoline (10, 30, 50, 100, 300, 500 μmol/L)	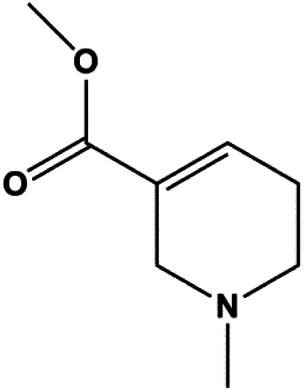	p53, Bax	Bcl-2	MCF-7	Feng et al. (2016)
Amygdalin (10, 20, 40 mg/mL)	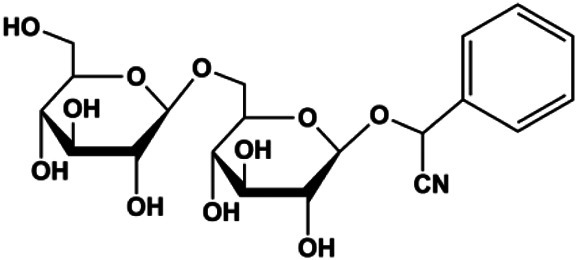	Bax, activated caspase-3, cleaved PARP, p-p38	Bcl-2	Hs578T	Lee and Moon, (2016)
Osthole (25, 50, 100 μmol/L)	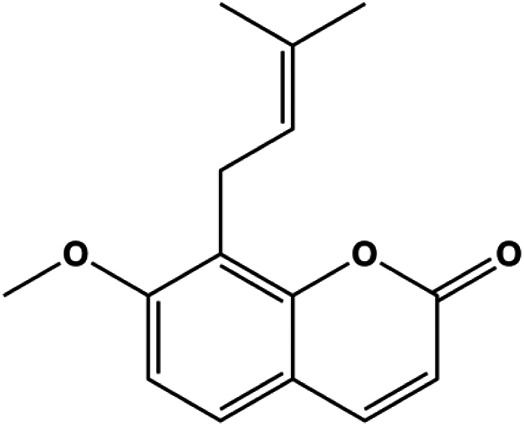	Bax, p53, p21, CytoC	Bcl-2	MCF-7	Zhang and Zhu, (2016)
Cordycepin (50, 100 μM)	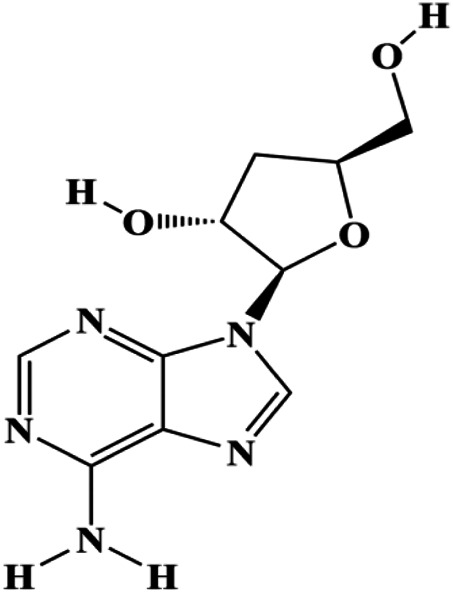	Cleaved caspase-8, cleaved caspase-9, cleaved caspase-3, Bax	Bcl-2	MCF-7 MDA-MB-231	Wang et al. (2016)
Violacein (0.45, 4.5 μM)	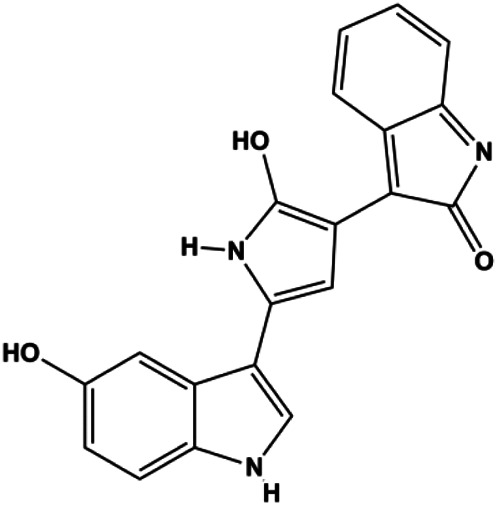	Bax, p53, caspase-3, Fas, FADD	Bcl-2, MDM2	MCF-7	Alshatwi et al. (2016)
Berberine (5, 10, 20, 40 µg/mL)	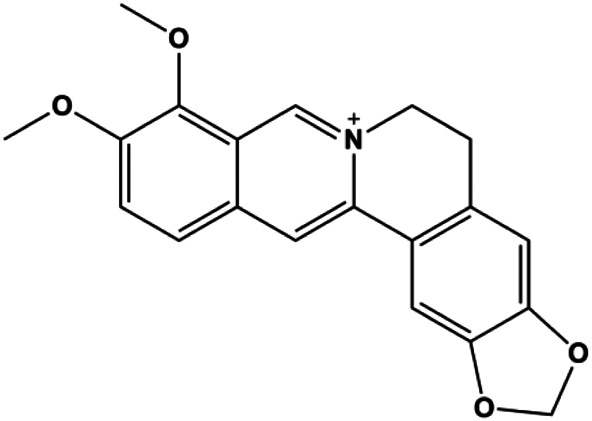	Cleaved caspase-3, cleaved caspase-9, Bax, CytoC	Bcl-2	BT549 MDA-MB-231	Zhao et al. (2017)
Liriodenine (0.1, 1, 10 µM)	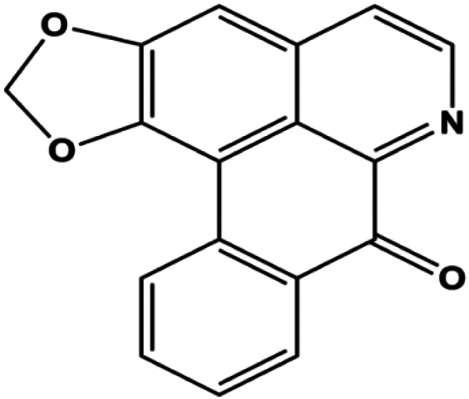	p53	Bcl-2, cyclin D1	MCF-7	Li et al. (2017b)
Polyphyllin I (8 μM)	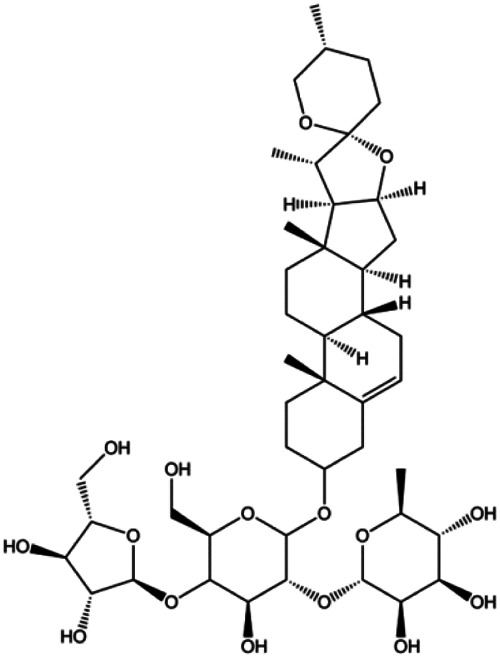	Cleaved caspase-9, cleaved caspase-3, cleaved PARP		MDA-MB-231	Li et al. (2017a)
Clematis hederagenin saponin (0.08, 0.4, 2, 10 µg/mL)	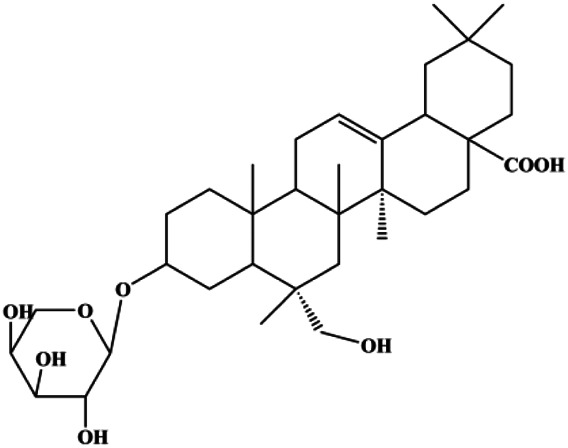	Caspase-3, caspase-9	Apaf-1, CytoC	MCF-7 MDA-MB-231	Cheng et al. (2018)
Kaempferol (50 μmol/L)	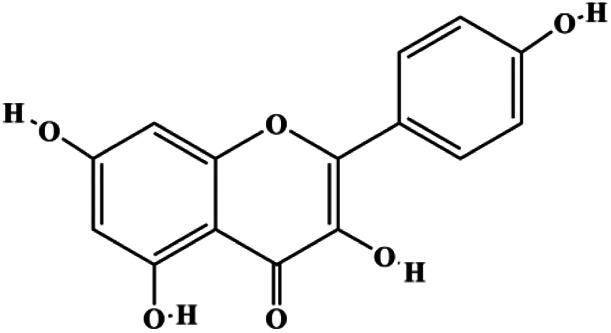	Cleaved caspase-9, cleaved caspase-3, p-A TM		MDA-MB-231 cells	Zhu and Xue, (2019)
Diosgenin (5, 10, 20, 40 µM)	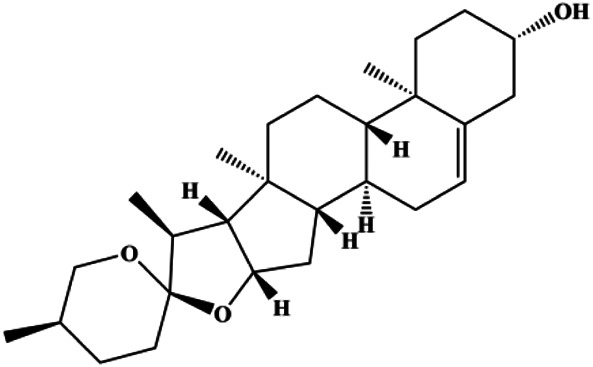	CytoC, cleaved caspase-9	Bcl-2	MCF-7 Hs578T	Liao et al. (2019)
Baicalein (50, 100 µM)	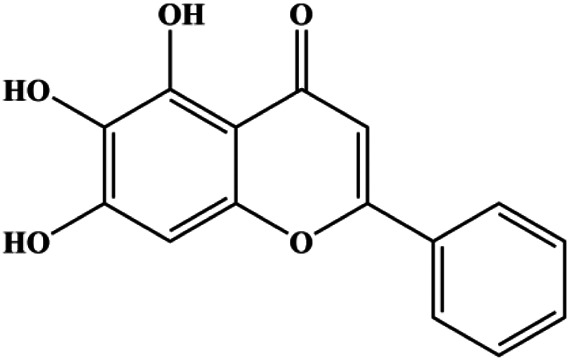	Bax, CytoC, cleaved caspase-9, cleaved caspase-3	Bcl-2	MCF-7	Liu et al. (2019)
Allicin (20, 45 µM)	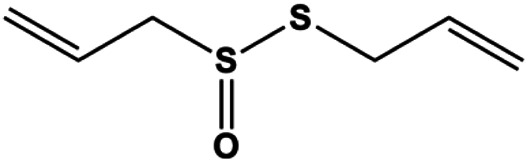	Activated caspase-3, activated caspase-8, activated caspase-9 NOXA, p21, Bak	BclXL	MCF-7 HCC-70	Rosas-Gonzalez et al. (2020)
Diosmetin (5, 10, 20, 30, 40, 50, 60, 70 μm/L)	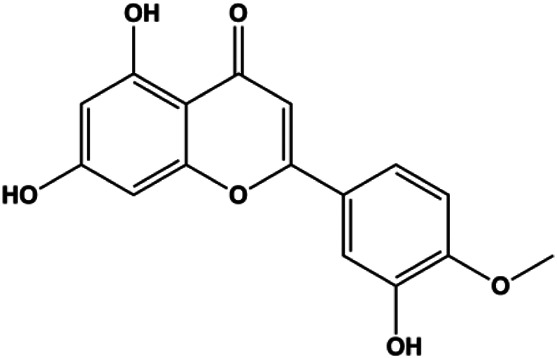	p53, Bax, caspase-3	Bcl-2	MCF-7	Wang et al. (2020)
Arnidiol (60 μM)	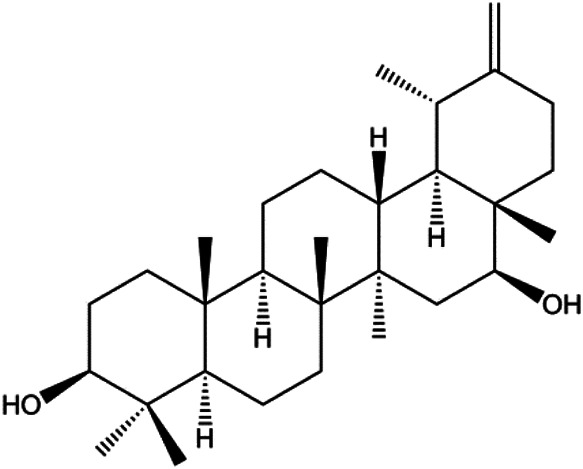	PARP, cleaved caspase-3		MDA-MB-231	Hu et al. (2020)
Catechol (10, 40, 80 μM)	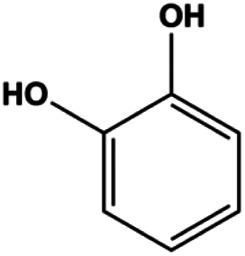	p-H2AX, p-ATM, p-ATR, cleaved caspase -9, cleaved caspase-3, Bax	BclXL, Bcl-2	MDA-MB-231	Vazhappilly et al. (2021)
Resveratrol (12.5, 25, 50, 100, 200 μM)	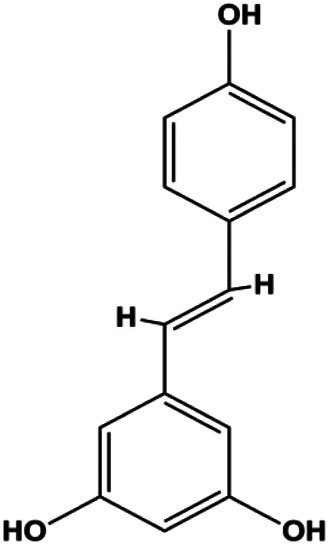	Cleaved PARP1, cleaved caspase-3	PCNA, Bcl-2	MDA-MB-231	Liang et al. (2021)
Cepharanthine (2 μM)	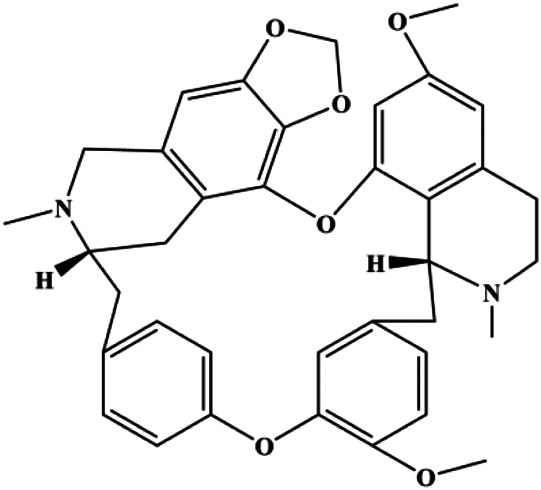	Cleaved caspase-3, cleaved PARP		MDA-MB-231	Shen et al. (2021)
Dehydrocostuslactone (5, 10, 20, 30, 40, 50, 60, 80, 100 μmol/L)	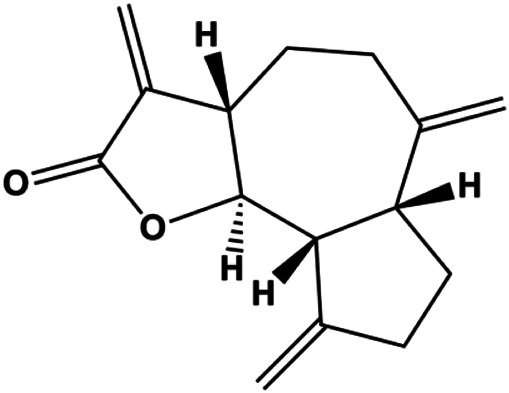	Bax, caspase-3, cleaved caspase-3	Bcl-2	SK-BR-3	Ma et al. (2021)
**FasL-Fas–mediated apoptosis**					
Icariside II (25, 50, 75 µM)	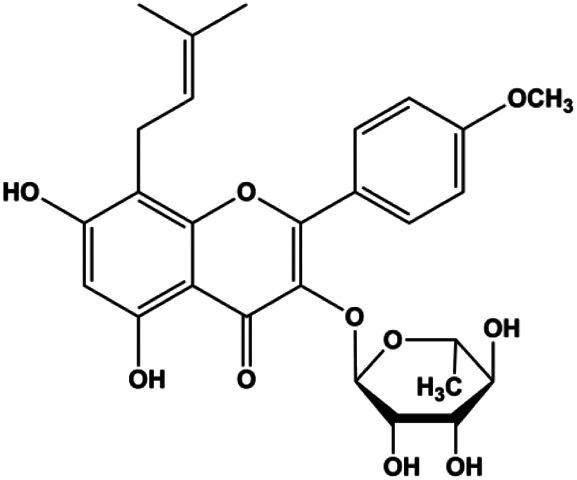	CytoC, cleaved caspase-3, cleaved PARP, cleaved caspase-7, cleaved caspase-8, Fas, FADD, BclXL, Bax, Bim		MCF7	Huang et al. (2012)
Gambogenic acid (0.4, 0.6, 0.8, 1.0 µg/mL)	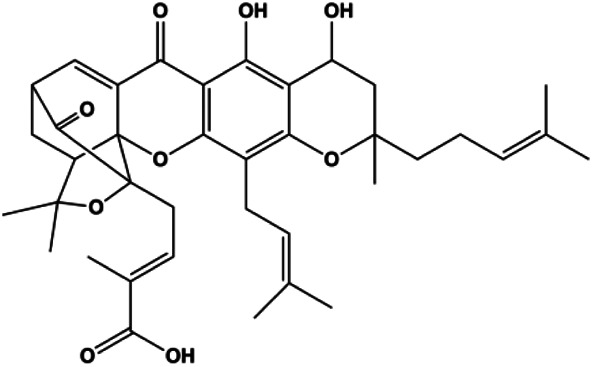	Fas, cleaved caspase-3, cleaved caspase-8, cleaved caspase-9, Bax	Bcl-2	MDA-MB-231	Zhou et al. (2013)
Genistein (0, 5, 10, 20 μmol/L)	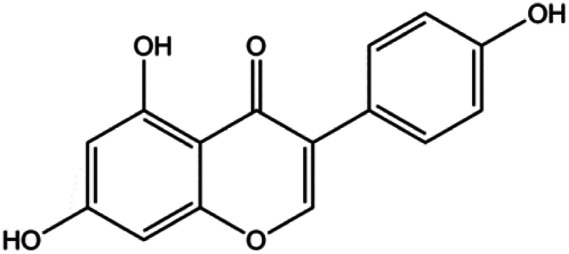	FADD, cleaved caspase-8, FasL		MDA-MB-231	Wang et al. (2013a)
α-Mangostin (1, 2, 3, 4 μM)	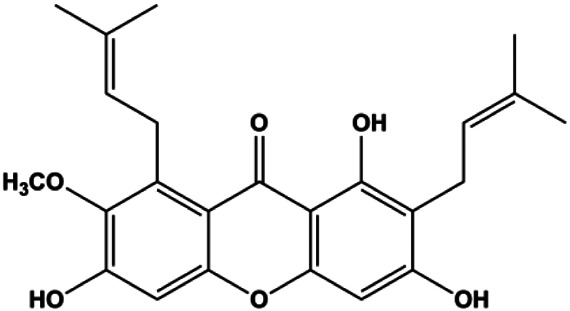	Cleaved PARP, Bax	Fas, Bcl-2, p-Fak	MCF-7 MDA-MB-231	Li et al. (2014)
Fraxetin (20, 40, 60 µM)	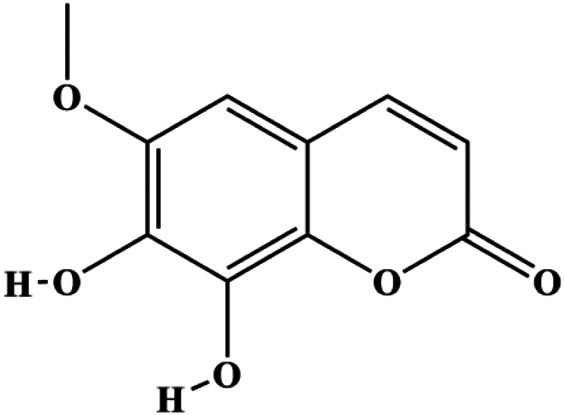	Fas, FasL, Bax	Bcl-2	MCF-7	Liu et al. (2017a)
Pulveraven A (50,100 µM)	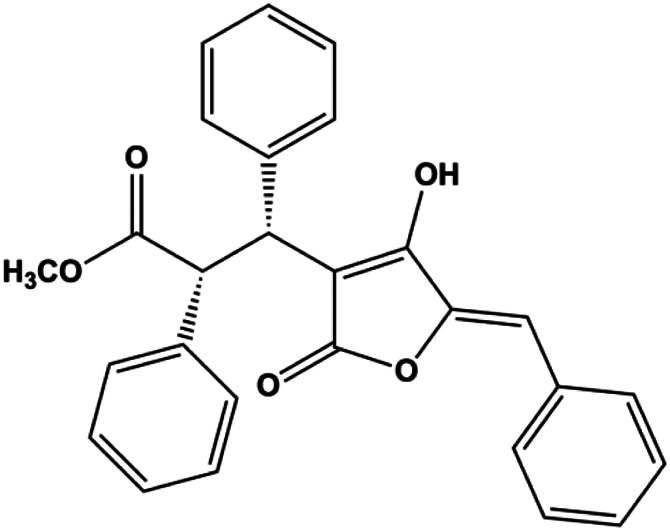	FADD, cleaved caspase-7, cleaved caspase-8, cleaved PARP, Bax	Bcl-2	MCF-7	Lee et al. (2021)
**PI3K/AKT pathway–mediated apoptosis**					
18β-Glycyrrhetinic acid (100 µM)	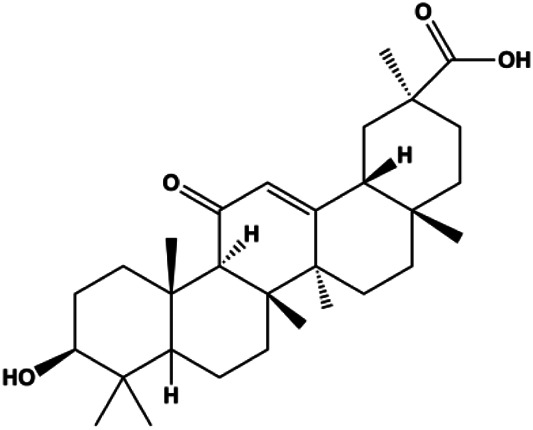	CytoC, activated caspase-9, Bax	Bcl-2	MCF-7	Sharma et al. (2012)
Fangchinoline (5, 10, 20 μmol/L)	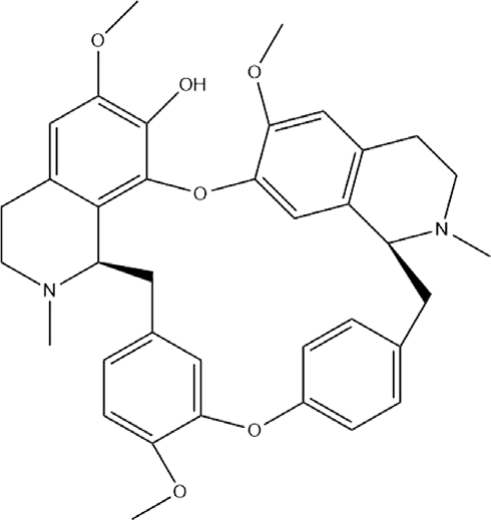		p-PI3K, p-Akt, p-mTOR	MDA-MB-231	Zhang et al. (2017)
Cucurbitacin E (100–200 nM)	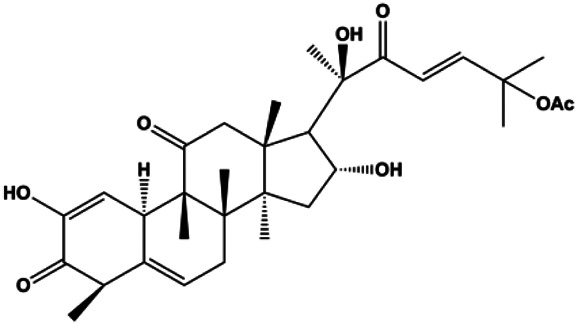	Cleaved caspase-3, cleaved PARP	Cyclin D1, survivin, Mcl-1, XIAP, Bcl-2, p-STAT3, p-ERK, p-AKT	MDA-MB-468	Kong et al. (2014)
Lycopene (100 μM)	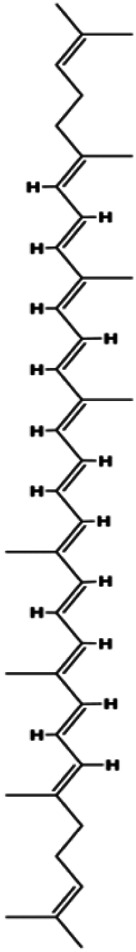	Cleaved PARP, p21, Bax	p-Akt, p-mTOR, cyclin D1	MDA-MB-468	Takeshima et al. (2014)
Piperlongumine (5, 10, 15, 20, 25 μM)	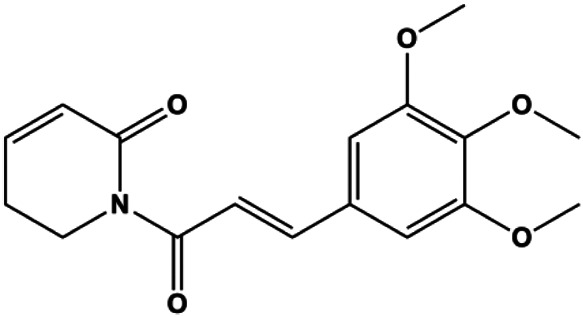	Bax, CytoC	p-Akt, p70S6K1, 4EBP1, cyclin D1, Bcl-2, p53	MDA-MB-231	Shrivastava et al. (2014)
d-Rhamnose β hederin (20, 30, 40 μg/mL)	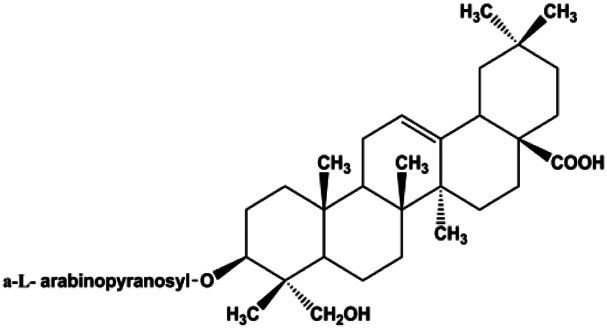		p-PI3K, p-Akt	MDA-MB-231 MCF-7	Cheng et al. (2014)
Genistein (0, 20, 40, 80 μM)	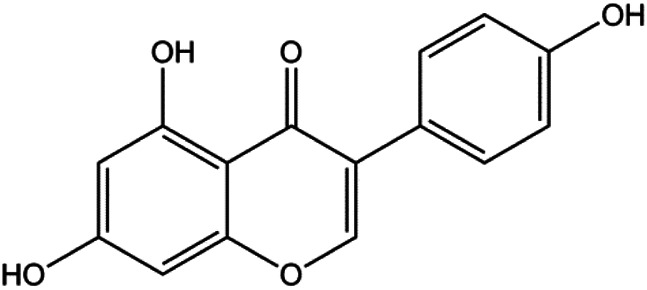	Bax	p-Akt, Bcl-2	MCF-7	Chen et al. (2015b)
Isorhamnetin (10 μM)	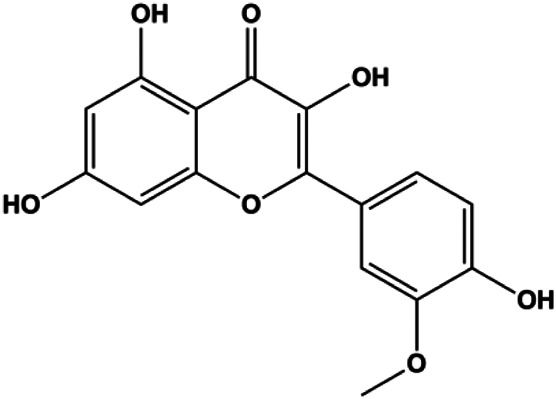	Bax, cleaved caspase-3	p-Akt, p-mTOR, p-MEK1/2, p-ERK1/2, Bcl-2, BclXL	MCF7 MDA-MB-468	Hu et al. (2015)
Calycosin (80 μM)	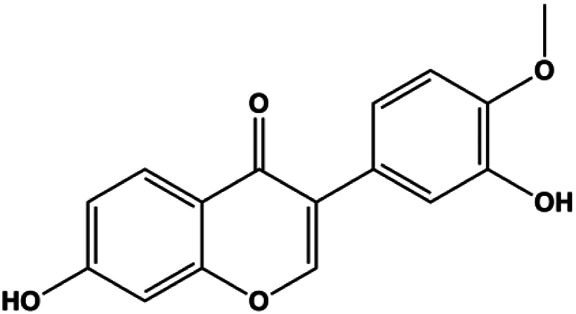		p-Akt, HOTAIR	MCF-7	Chen et al. (2015c)
Ramentaceone (5, 10, 15 μM)	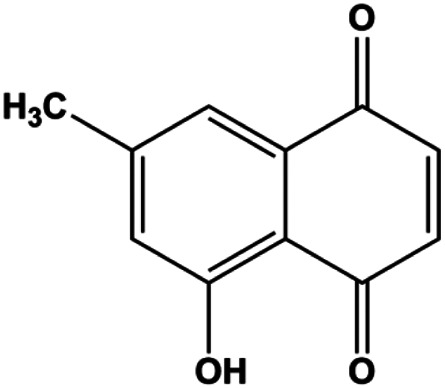	Bax, Bak, cleaved caspase-3, cleaved PARP	PI3K p85, p-PI3K, p-Akt (Ser473), Bcl-2	BT47 SKBR3 MCF-7 MDA-MB-231	Kawiak and Lojkowska, (2016)
Ginsenoside Rg3 (100 ng/mL)	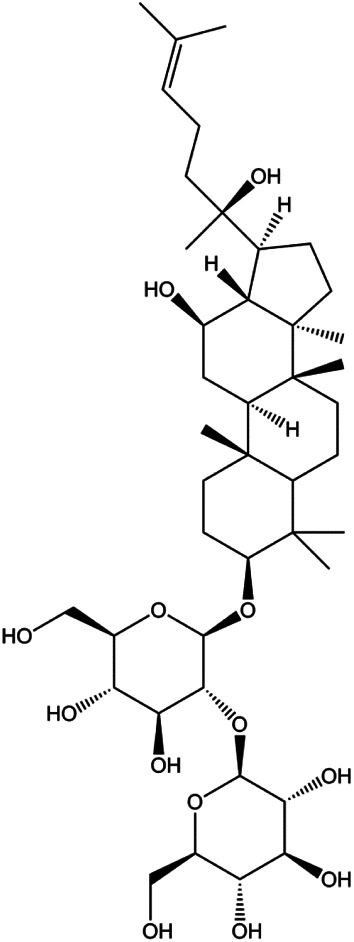		p-Akt, MGBA	MDA-MB-231	Peng et al. (2016)
α-Mangostin (15, 30 μM)	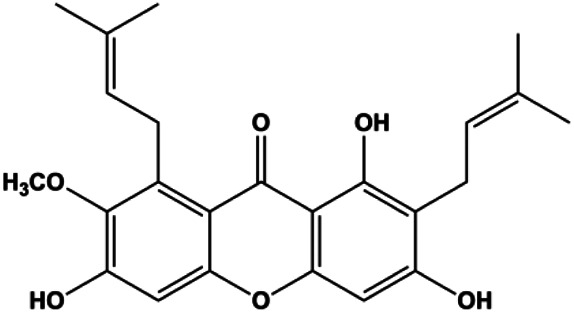	Cleaved caspase-3, cleaved caspase-9	Bcl-2, Mcl-1	T47D	Kritsanawong et al. (2016)
Cepharanthine (with 10 µM in MCF-7 cells, with 6 µM in MDA-MB-231 cells)	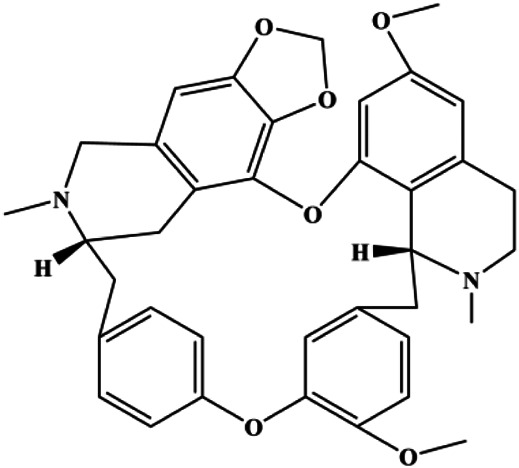	Bax, Bad, cleaved caspase-3, cleaved caspase-9	Bcl-2	MCF-7 MDA-MB-231	Gao et al. (2017)
Baicalein (10, 20, 40 µM)	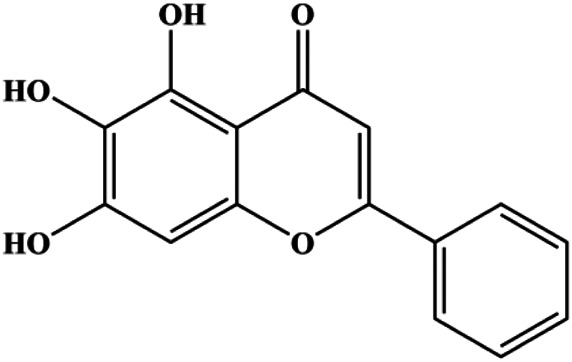	IκB	p-Akt, p-mTOR, NF-κB, p-IκB	MCF-7 MDA-MB-231	Yan et al. (2018)
Fisetin (20μM, 40μM, 80 μM)	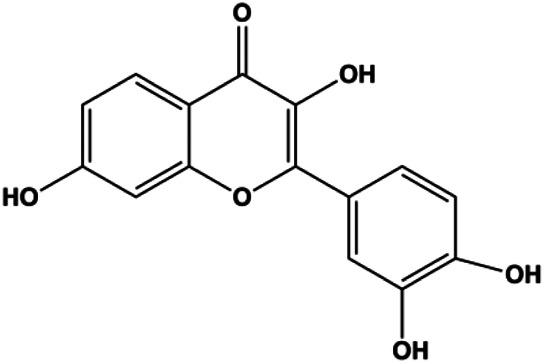	Bax, caspase-3, caspase-8, caspase-9	p-PI3K, p-Akt, p-mTOR, p-P70	4T1	SUN et al. (2018)
Stachydrine hydrochloride (5, 20, 50, 100, 200 µM)	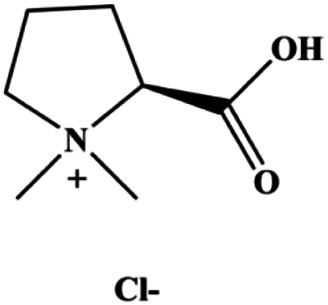	Cleaved caspase-3	Bcl-2, p-Akt, p-ERK	MCF-7 T47D	Wang et al. (2017)
Paris saponins (XA2) (1.25, 2.5, 5 µM)	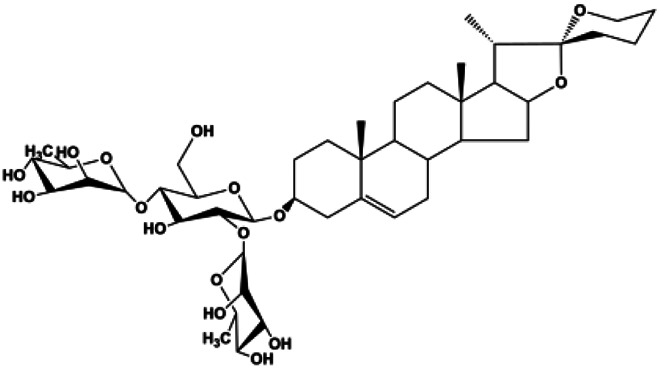		p-Akt, p-mTOR, p-P70S6K	MCF-7 MDA-MB-231	Xie et al. (2017)
Ginsenoside Rg5 (10, 20 mg/kg)	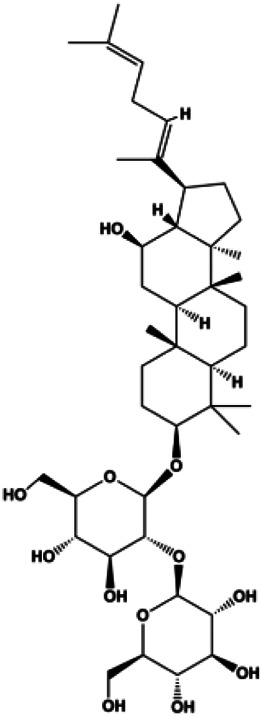	Ras, cleaved caspase-8, cleaved caspase-9, Bax, CytoC	Bcl-2	MCF-7	Liu and Fan, (2018)
20(S)-protopanaxadiol (15, 30, 60 µM)	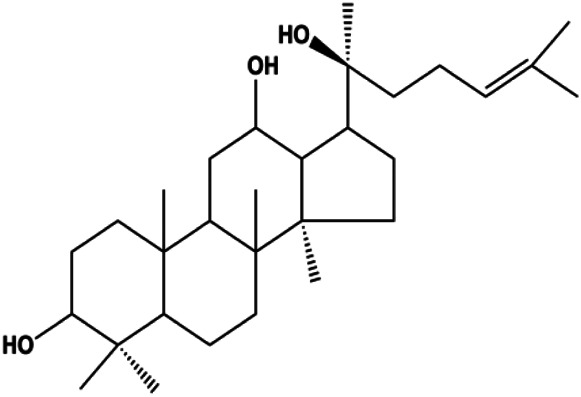	p-PTEN, p53	p-Akt (Thr308), p-Akt (Ser473), p-mTOR (Ser2448), p-FoxO1 (Ser256), p-MDM2 (Ser166), p-NF-κB, p65 (Ser536), p-GSK-3β (Ser9), p27kip1, CytoC	MCF-7	Zhang et al. (2018a)
Galangin (20, 40, 80, 160 μmol/L)	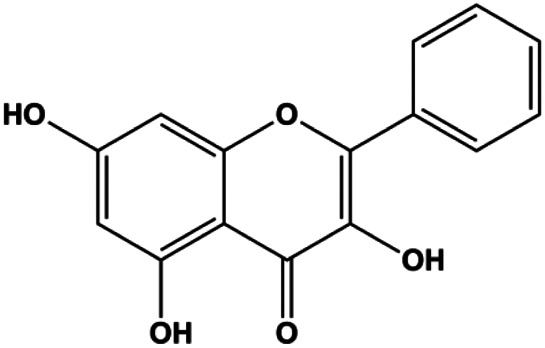	Bax, cleaved caspase-9, cleaved caspase-8, cleaved caspase-3, cleaved Bid, Bad, p21, p27, p53	Bcl-2, p-PI3K, p-Akt, cyclin D3, cyclin B1, CDK1, CDK2, CDK4	MCF-7	Liu et al. (2018b)
Tetramethylpyrazine (800, 1,600, 3,200 µM)	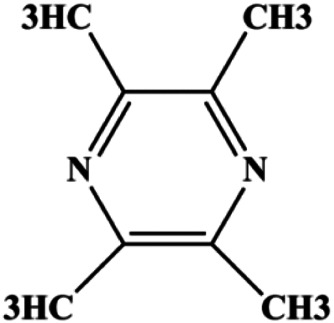	Cleaved caspase-3	p-Akt	MDA-MB-231	Shen et al. (2018)
Daucosterol linoleate (25, 50, 100 mg/mL)	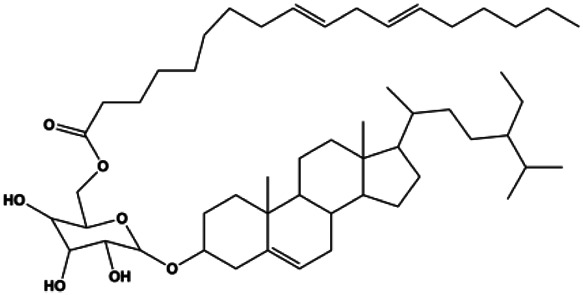	Cleaved caspase-3, cleaved caspase-7, cleaved caspase-8, cleaved caspase-9, cleaved PARP-1, Bax, Bad	BclXL, Bcl-2, XIAP	MCF-7	Han et al. (2018)
Cannabidiol (1–7 µM)	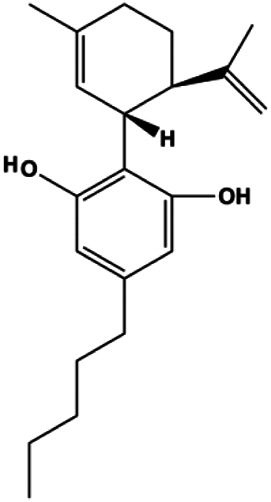	PPARγ	mTOR, cyclin D1	MDA-MB-231 T-47D	Sultan et al. (2018)
Crambescidin 800 (0.01, 0.1, 0.5, 1, 5, 10,15, 20 µM)	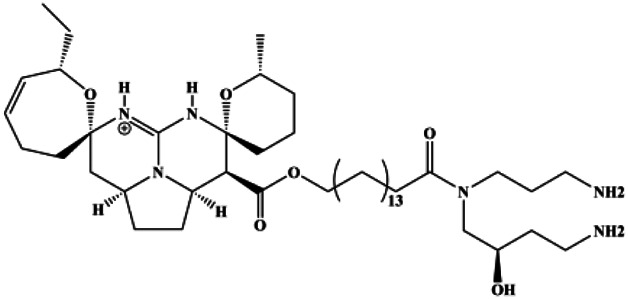	p21	Cyclin D1, CDK4, CDK6	T11 SUM159PT	Shrestha et al. (2018)
Eupatorin (5 μg/mL)	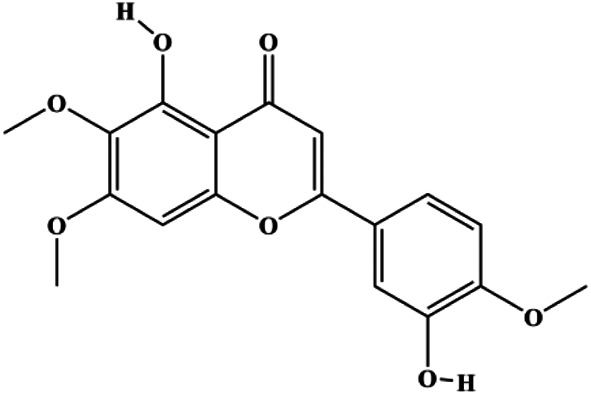	Bak1, HIF1A, Bax, Bad, CytoC, SMAC/Diablo, activated caspase-9, activated caspase-8, Rb-Raf-1, GSK-3β	Akt (pan), p-PDK1	MDA-MB-231	Razak et al. (2019)
Kaempferol (25, 50, 100 μmol/L)	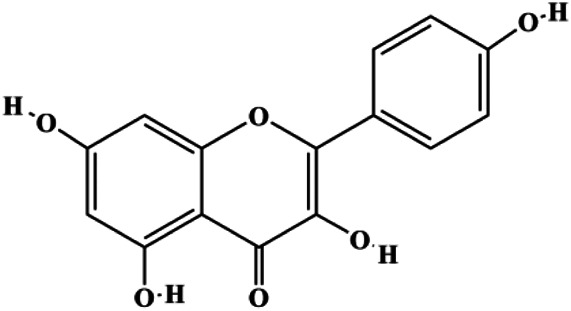		p-PI3K, p-AKT, p-GSK-3β	SUM190	Zhao et al. (2019)
Curcumin (10, 30 µM)	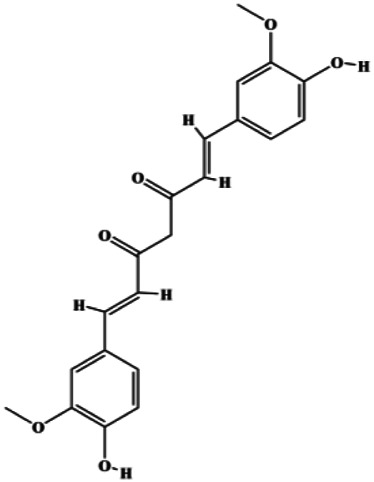	p21, Bax, cleaved caspase-3	CDC25, CDC2, Bcl-2	T47D MCF-7	Hu et al. (2018)
Ginsenoside Rk1 (40, 80, 120 µM)	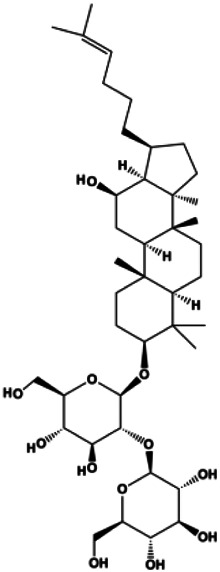	Bax, CytoC, cleaved caspase-3, cleaved caspase-8, cleaved caspase-9	Bcl-2	MDA-MB-231	Hong and Fan, (2019)
Parthenolide (5, 10 μmol/L)	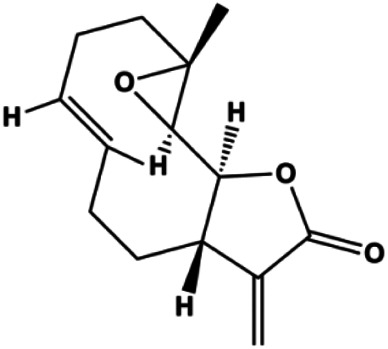	Caspase-3	Bcl-2	MDA-MB-231 MCF-7	Han et al. (2019)
Flavopereirine (5, 10, 15 μM)	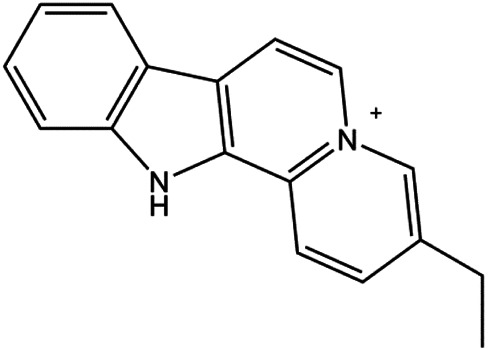	Cyclin D1, cyclin E1, p21, 27, activated caspase-3, activated caspase-9, cleaved PARP, p-ERK1/2, p38	Cyclin A2, CDK-2, BclxL, cleaved caspase-8	MDA-MB-231	Yeh et al. (2019)
Avicularin (6.25, 12.5, 25, 50, 100 μmol/L)	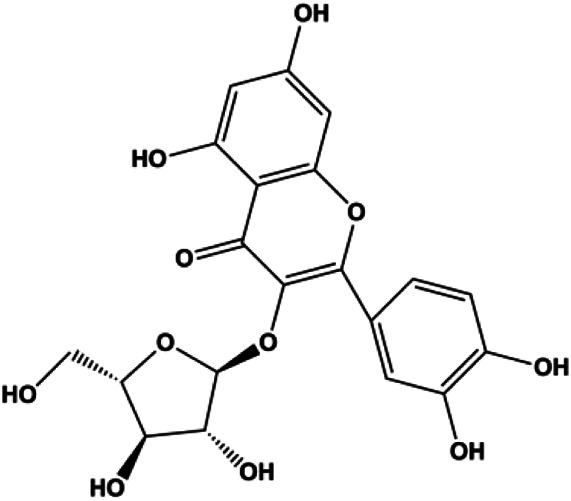		PI3K, p-PI3K, AKT, p-Akt, Bcl-2, BclXL, CDK2	MDA-MB-231	Yu et al. (2020)
Magnoflorine (1.25, 2.5, 5 μM) and Doxorubicin (0.1, 0.25, 0.5, 1 μM)	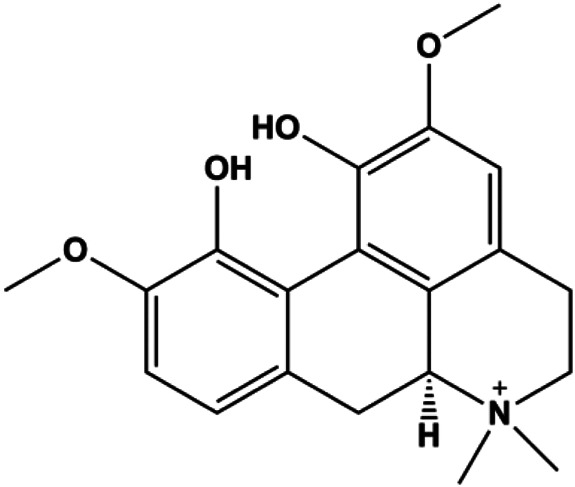	p21, p53, cleaved caspase-9, cleaved caspase-3, Beclin-1, LC3-I	CDK1, CDK2, cyclin B1, Bcl-2, p62	MCF-7 MDA-MB-231	Wei et al. (2020)
Erianin (40, 80, 160 nM)	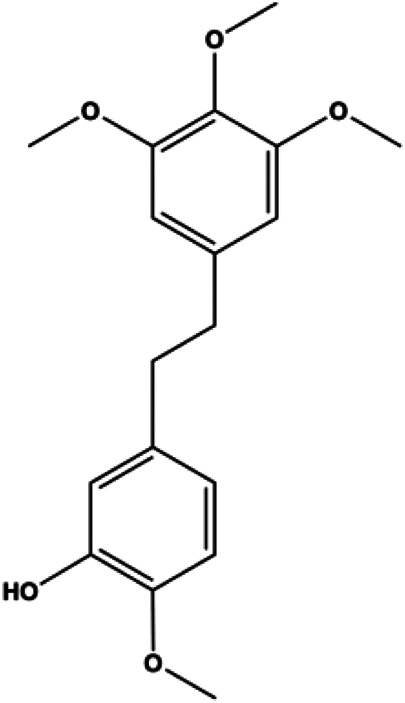	p21, p27, PARP, Bax, caspase-3, caspase-9	CDK1, cyclin B1, p-PI3K, p-Akt	MDA-MB-231 EFM-192A cell	Xu et al. (2021)
**MAPK pathway–mediated apoptosis**					
Formononetin (25, 50, 100 µM)	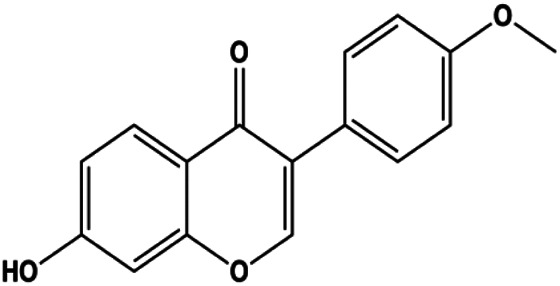	Bax, Ras, Raf, p-p38	Bcl-2	MCF-7	Chen and Sun, (2012)
Genipin (0.05, 0.1, 1 mM)	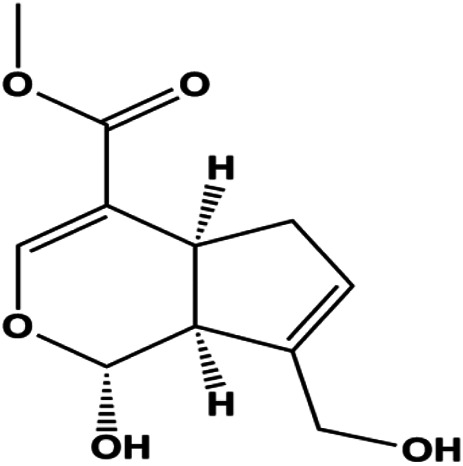	Bax, activated caspase-3, p38, p-JNK	Bcl-2	MDA-MB-231	Kim et al. (2012)
Tetramethylpyrazine (0.25, 0.50, 1.00, 1.50, 2.00 mg/mL)	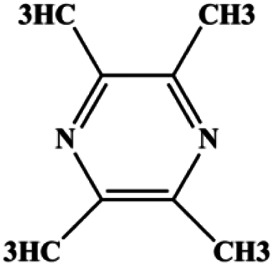	p-p38, p-JNK	p-ERK	MCF7	Lai et al. (2016)
Deoxypodophyllotoxin (10^1^–10^4^ ng/mL)	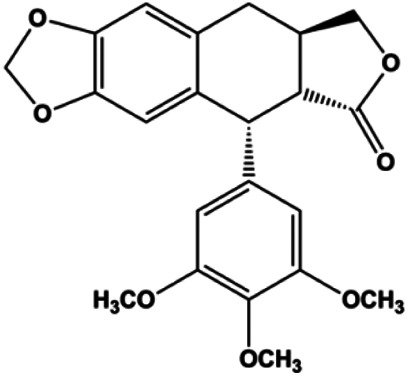	Cleaved caspase-3, ERK1/2, IkB-α	p-ERK	MB231	Benzina et al. (2015)
Emodin (20, 40, 80 μmol/mL)	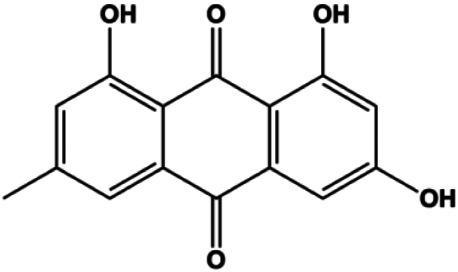	AP-1		MCF-7	Fan et al. (2015)
Quercetin (40 μM)	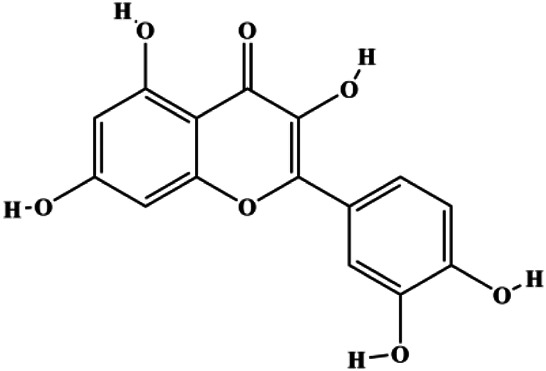	p21	Cyclin D1, Twist, p-p38	MCF-7	Ranganathan et al. (2015)
Quercetin (20 μM)	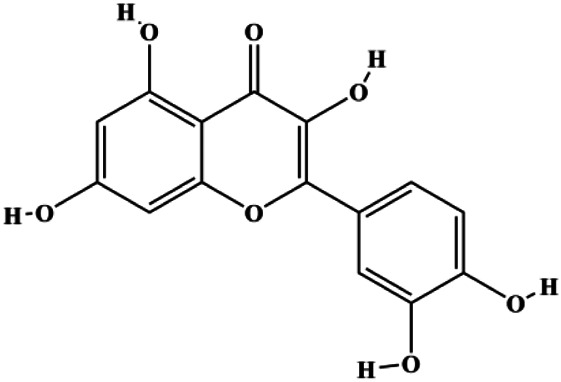	FasL, GADD45, p53, p21, p-JNK, translocation of Foxo3a		MDA-MB-231	Nguyen et al. (2017)
Isocryptotanshinone (2.5, 5, 10 μM)	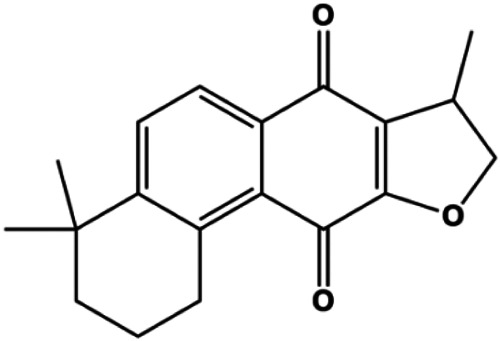	Bsx, Bak, cleaved PARP, cleaved caspase-3, cleaved caspase-9, p-JNK, p-ERK, p-p38	Bcl-2, BclXL	MCF-7	Zhang et al. (2015a)
Gallic acid (5, 25, 50 µM)	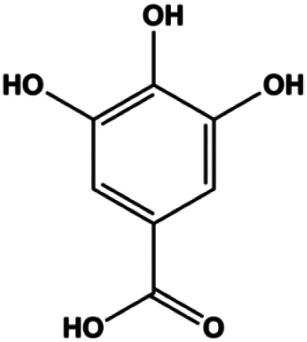	p21^Cip1^, p27^Kip1^, activated caspase-9, activated caspase-3	Cyclin D1/CDK4, cyclin E/CDK2	MDA-MB-231	Lee et al. (2017)
Sophoraflavanone G (5, 10, 15, 20, 30 µM)	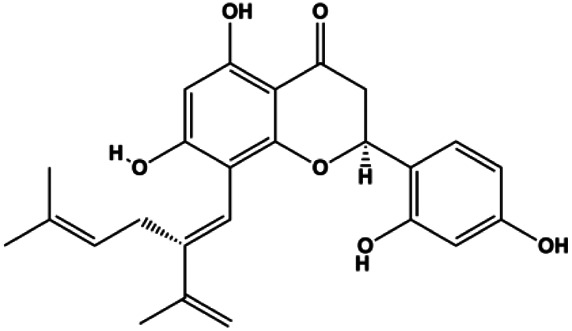	Cleaved caspase-8, cleaved caspase-3, cleaved caspase-9	Bcl-2, BclXL	MDA-MB-231	Huand et al. (2019)
Cardamonin (CD) (20 µM)	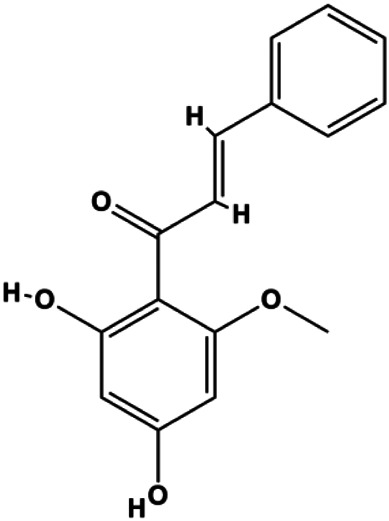	Foxo3a, p27, p21, Bim, p-JNK, cleaved caspase-3	Cyclin D1	MDA-MB 231 MCF-7	Kong et al. (2019)
Silymarin (100, 200 µg/mL)	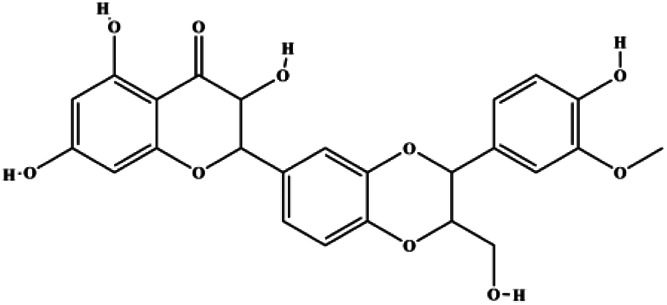	Bax, cleaved PARP, cleaved caspase-9, p-JNK	Bcl-2, p-p38, p-ERK1/2	MDA-MB-231 MCF-7	Kim et al. (2021)
**NF-κB–mediated apoptosis**					
Oridonin (20 µM)	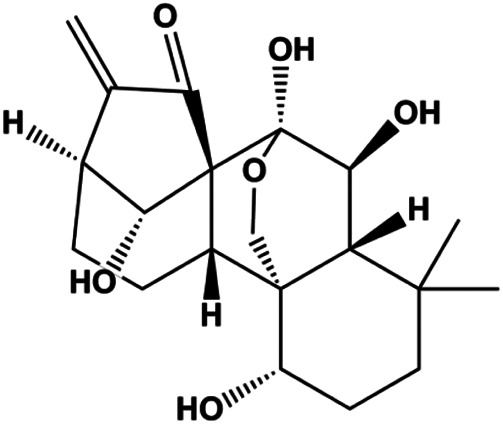	Bax, cleaved PARP, Fas, PPARγ	Bcl-2, caspase-8, NF-κB (p65), IKKα, IKKβ, p-mTOR	MDA-MB-231 MCF-7	Wang et al. (2013b)
Delphintin (10, 20, 40, 80, 160 μmol/L)	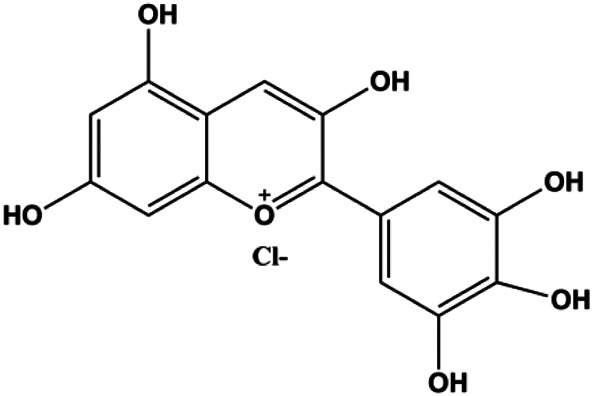	IκBα, IKKα, IKKβ, PKCα	p-NF-κB/p65, p-IκBα, p-IKKα/β, p-PKCα	MDA-MB-453 BT-474	Wu et al. (2018a)
Chrysophanol (5, 10, 20 µM)	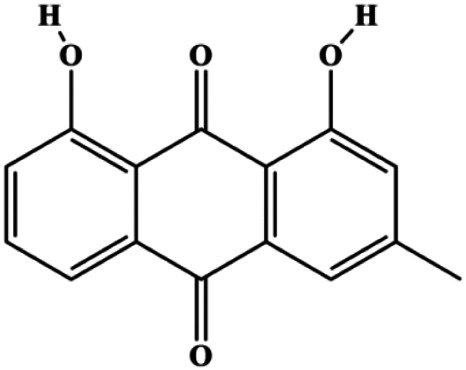	p27, cleaved caspase-3, cleaved PARP	Cyclin D1, cyclin E, Bcl-2, p65, IκB	MCF-7 MDA-MB-231	Ren et al. (2018)
Capsaicin (100, 150 μmol/L)	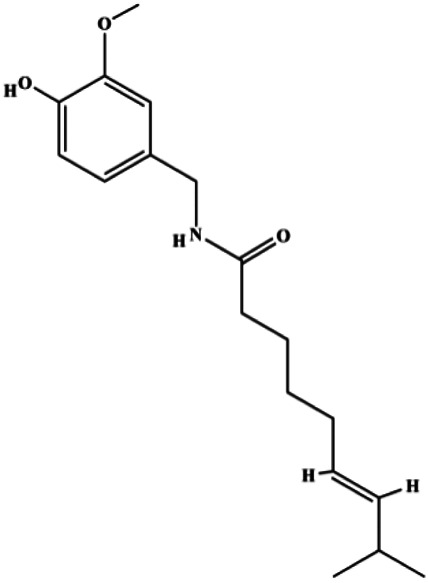	Bax, cleaved caspase-3	FBI-1, Ki-67, Bcl-2, survivin	MCF-7 MDA-MB-231	Chen et al. (2021)
**ROS-mediated apoptosis**					
Vernodalin (3.125, 6.25, 12.5 µg/mL)	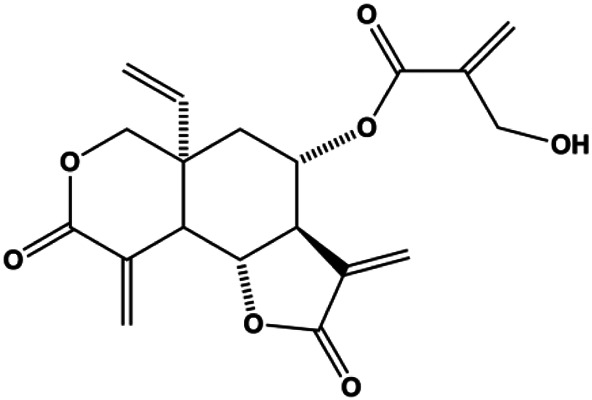	CytoC, cleaved PARP, caspase-3, cleaved caspase-7, cleaved caspase-9	Bcl-2, BclxL, MMP	MCF-7 MDA-MB-231	Looi et al. (2013)
Ziyuglycoside II (2.5, 10, 40 µM)	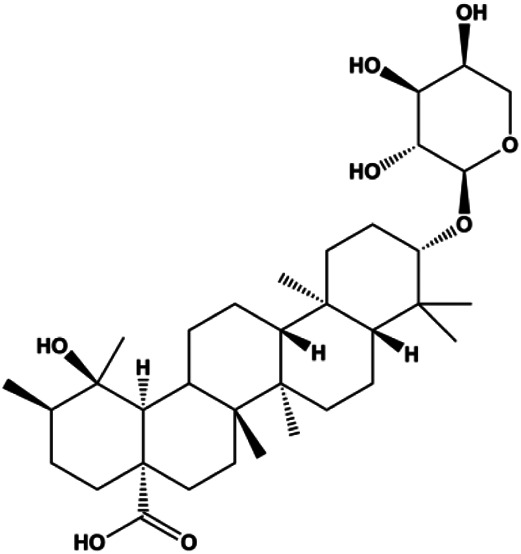	p21/WAF1, p-Cdc25C, p-Cdc2	Cdc25C, Cdc2, cyclin A, cyclin B1	MDA-MB-231 MCF-7	Zhu et al. (2014)
Fucoidan (0.2–1 mg/mL)	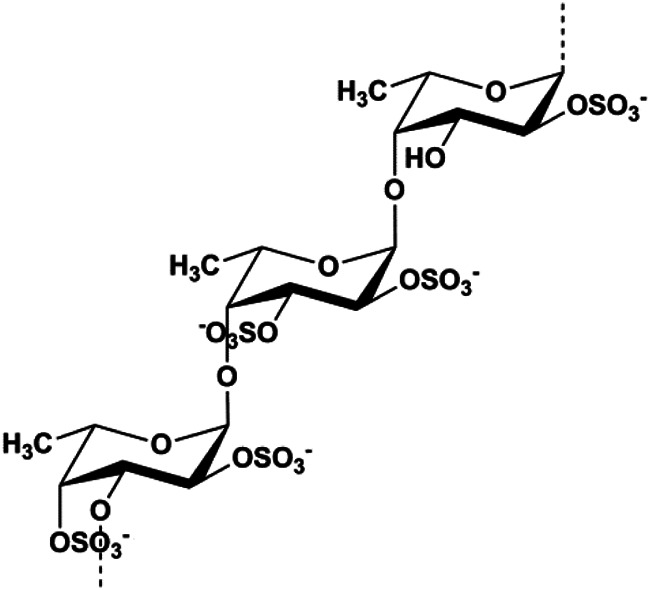	Caspase-8, CytoC, Bax	Cyclin D1, CDK-4, Bcl-2	MCF-7	Banafa et al. (2013)
Berberine (50 μM)	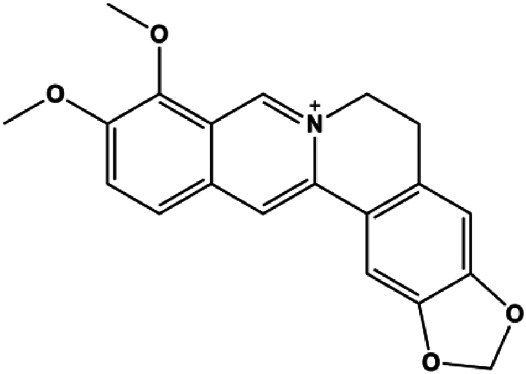	Bax, ROS, p-JNK, CytoC, AIF, activated caspase-3	Bcl-2	MDA-MB-231 MCF-7	Xie et al. (2015)
Geraniin (5, 10, 25 μM)	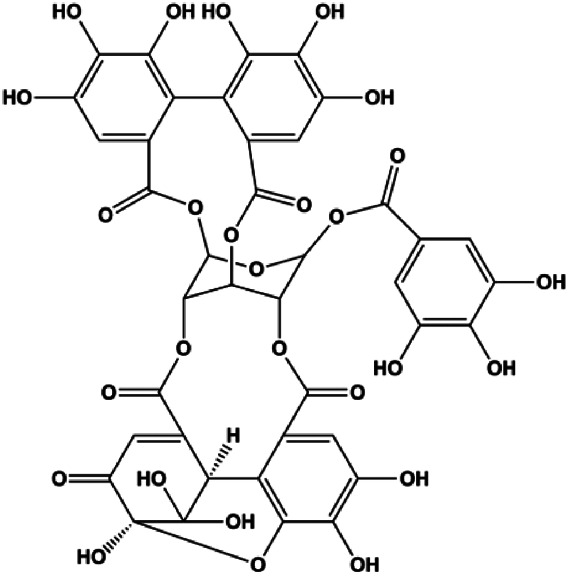	Cleaved PARP, cleaved caspase-3	Bcl-2, p-ERK1/2, p-Akt, p-p38	MCF-7	Zhai et al. (2016)
Hyperoside (25, 50, 100 µM)	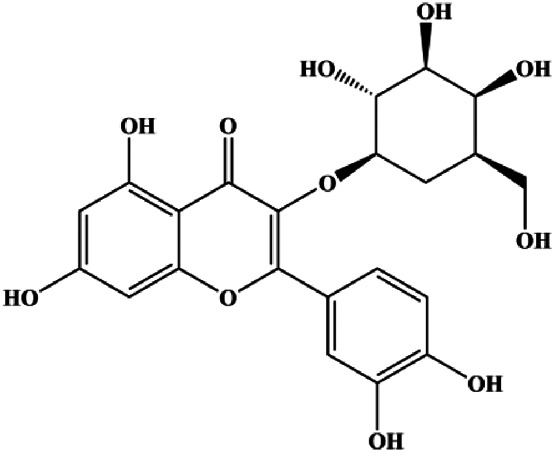	Bax, cleaved caspase-3	Bcl-2, XIAP	MCF-7 cells 4T1 cells	Qiu et al. (2019)
Crocin (0.5–6 mM)	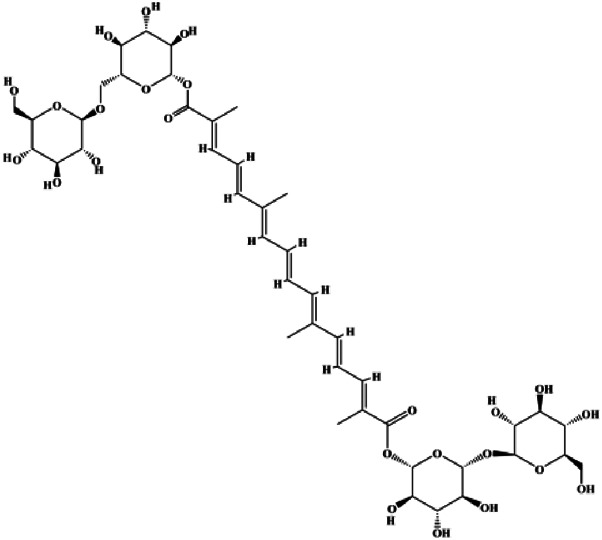	Foxo3a, Bim, PTEN, activated caspase-3, BAX	Bcl-2	MCF-7 MDA-MB-231	Nasimian et al. (2020)
Tetrandrine (51 μM)	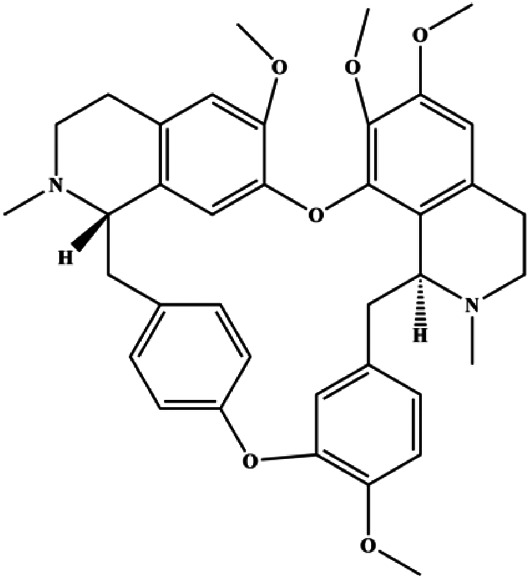	Activated caspase-8, activated caspase-9, activated caspase-3		MDA-MB-231	N et al. (2019)
(−)-Epicatechin (350 μM)	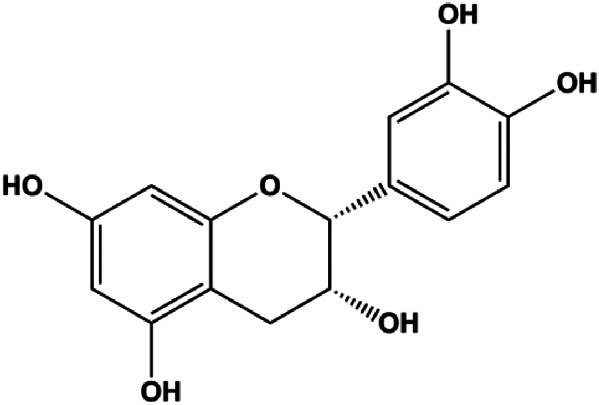	Bad, Bax		MCF-7 MDA-MB-231	Pereyra-Vergara et al. (2020)
Formononetin (8, 16, 32, 64, 128 μmol/L)	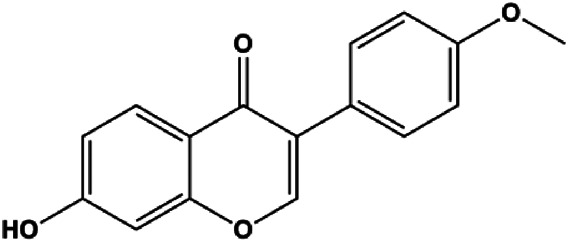	Bax, cleaved caspase-3	Bcl-2	MCF-7	Jia et al. (2020)
**JAK-STAT3–mediated apoptosis**					
Apigenin (0, 20, 40, 60 μM)	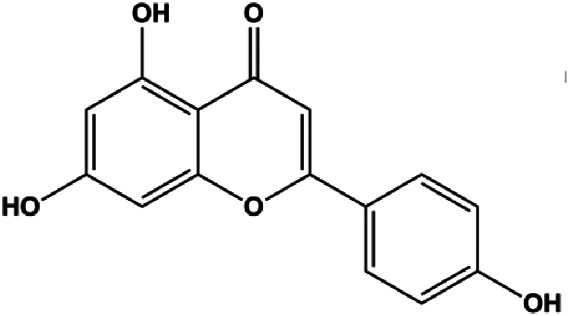	Cleaved caspase-8, cleaved caspase-3, cleaved PARP	p-JAK2, p-STAT3	MDA-MB-453 (HER2-overexpressing)	Seo et al. (2014)
Berberine (10, 20, 40 μg/mL)	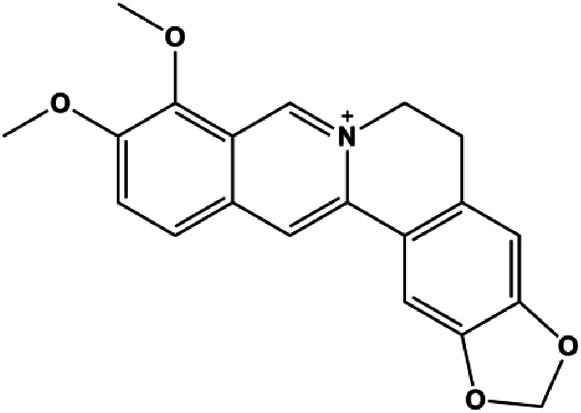	Bax, cleaved PARP, cleaved caspase-3	p-JAK2, p-STAT3, Bcl-2	MCF-7	Zhang et al. (2015b)
7β-(3-Ethyl-*cis*-crotonoyloxy)-1α-(2-methylbutyryloxy)-3,14-dehydro-Z-notonipetranone (2.5, 5, 10 µM)	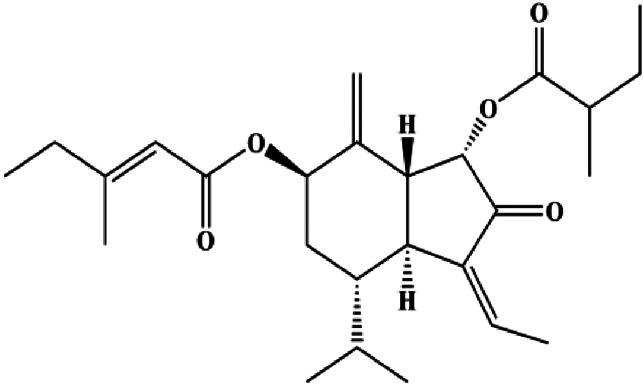	Cleaved caspase-3, cleaved caspase-8, cleaved PARP	p-STAT3 (Tyr705), p-JAK1, p-JAK2, p-Src, Bcl-2, COX-2, cyclin D1	MDA-MB-231	Jang et al. (2019)
Curcumol (0, 12.5, 25, 50, 100 μg/mL)	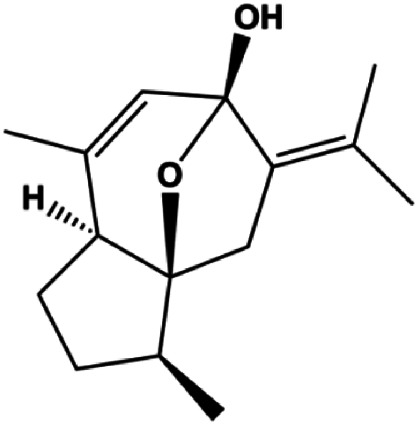		p-JAK2, p-STAT3	MCF-7	Ma et al. (2020)
Saikosaponin D (0, 5, 10, 20 μmol/L)	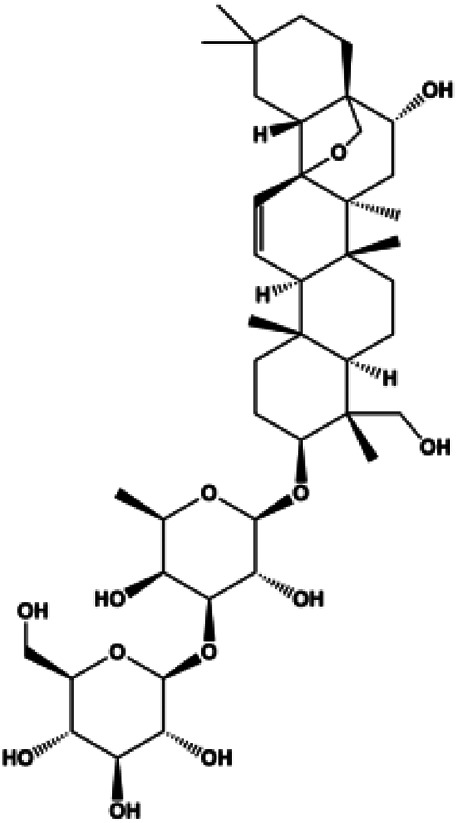		JAK2, STAT3, Ki-67	MCF-7	Feng et al. (2021)
**Endoplasmic reticulum stress–mediated apoptosis**					
Fucoxanthin (10, 20, 40 μmol/L)	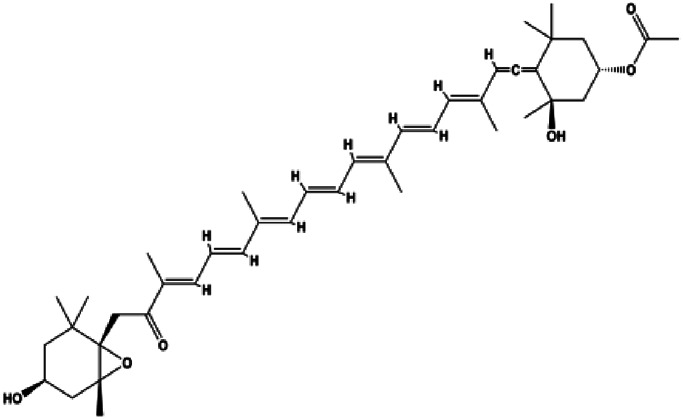	Bax, activated caspase-4, activated caspase-7, activated caspase-9, CytoC	Bcl-2	MCF-7	Hou et al. (2014)
Oleandrin (with 14.5 nM in MCF-7, with 24.62 nM in MDA-MB-231)	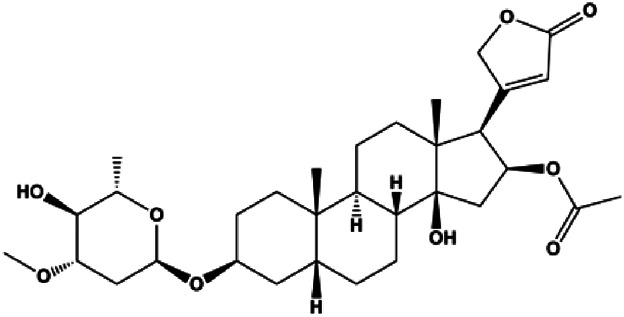	Bax, Bim, IF2α, ATF4, CHOP, p-PERK, p-eIF2α	Bcl-2	MCF-7 MDA-MB-231	Li et al. (2020)
Salviaflaside (0.1, 1, 10 μmol/L)	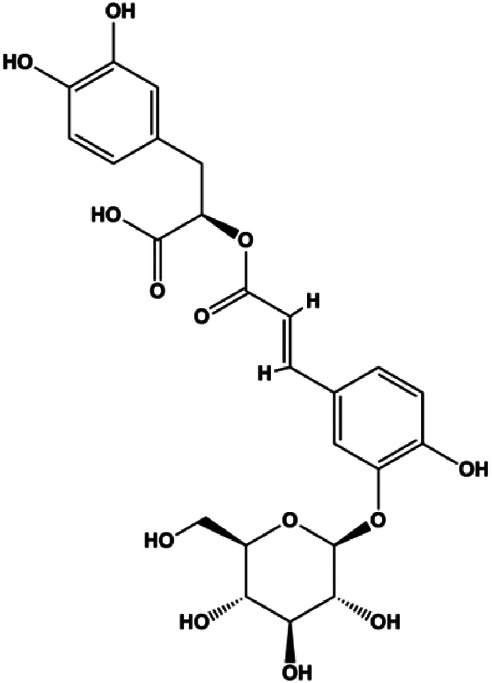	GRP78, ATF4, CHOP, p-PERK, p-eIF2α, cleaved caspase-3	Bcl-2/Bax	MDA-MB-231	Wu et al. (2018b)
**Other pathways–mediated apoptosis**					
Curcumin (30 µmol/mL)	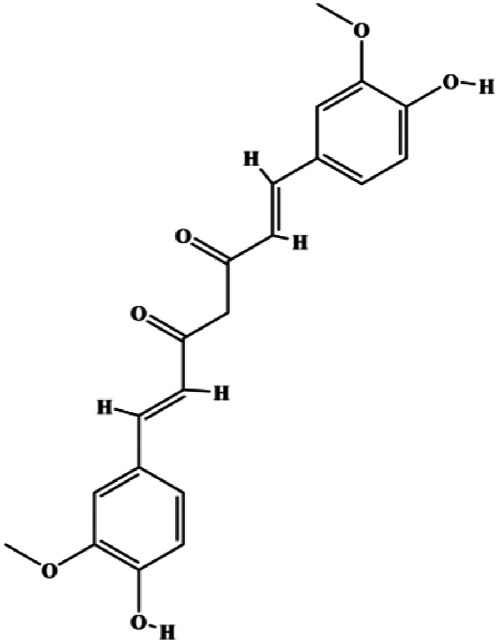		pERK1/2, pEGFR	MDA-MB-231	Sun et al. (2012)
Ursolic acid (10, 20, 30 µmol/L)	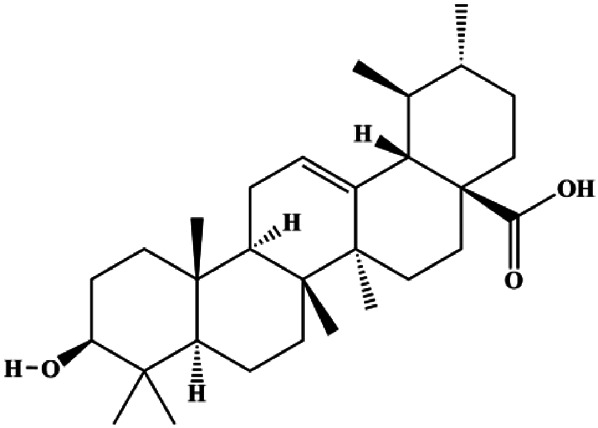		FOXM1, cyclin D1, CDK4	MCF-7	Wang et al. (2012)
Triptolide (10, 25, 50 nM)	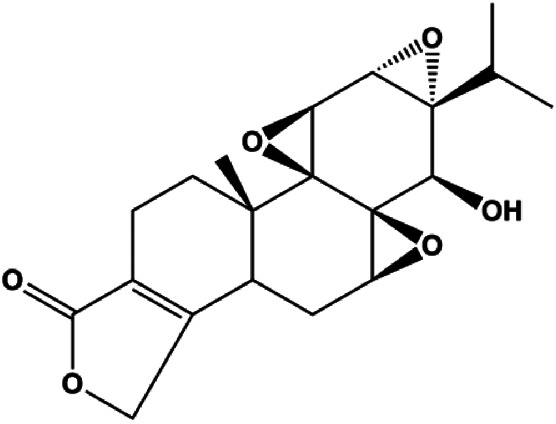		β-Catenin	MDA-MB-231 BT-474	Shao et al. (2014)
Calycosin (10, 50, 100 μL)	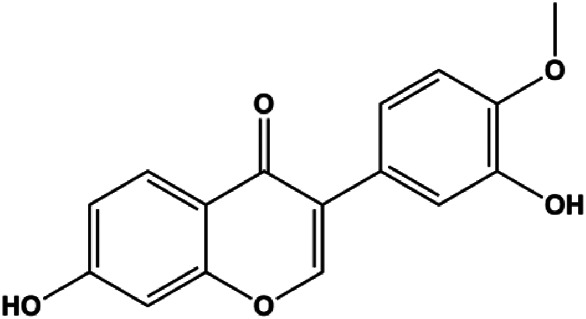	p53, Cleaved caspase-3	SIRT1	MCF-7	Fang et al. (2015)
Gambogic acid lysinate (0.25, 0.50, 1.00, 2.00, 4.00, 8.00 μmol/L)	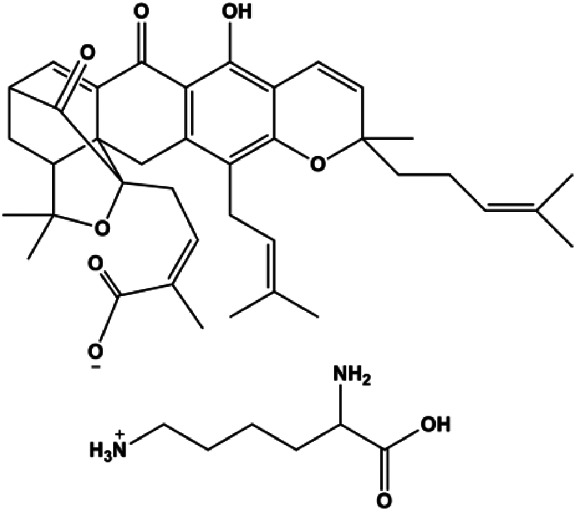	Cleaved caspase-3	SIRT1	MCF-7	Wei et al. (2015)
Icariin (10, 25, 50, 75 µM)	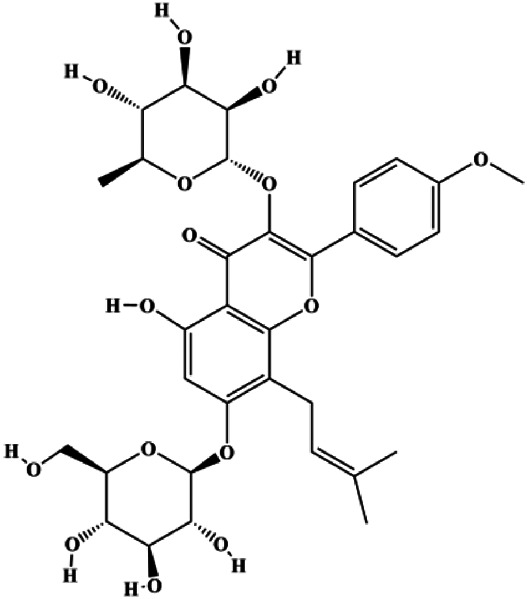	Caspase-3, PARP, p62	CDK2, CDK4, cyclin D1, Bcl-2, LC3-1, LC3-II, AGT5, Beclin-1	MCF-7/TAM	Cheng et al. (2019)
Ellagic acid (1–10 μM)	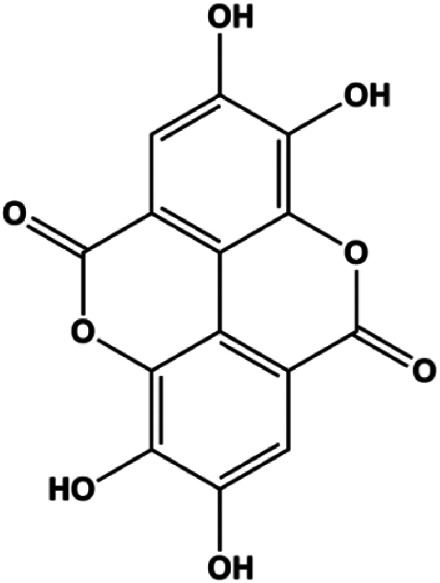		CDK6	MCF-7 MDA-MB-231	Yousuf et al. (2020)
Flavonoid calycopterin (150 μM)	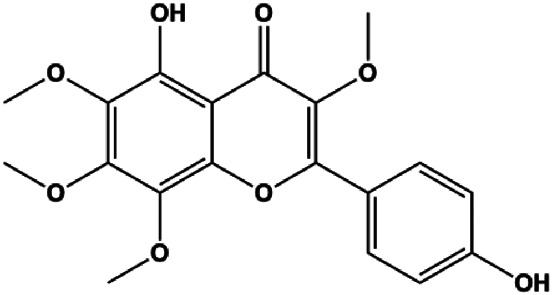	Bax, caspase-3, caspase-8	Bcl-2	MDA-MB-231	Moradi et al. (2020)
Quercetin (40, 80, 160 μmol/L)	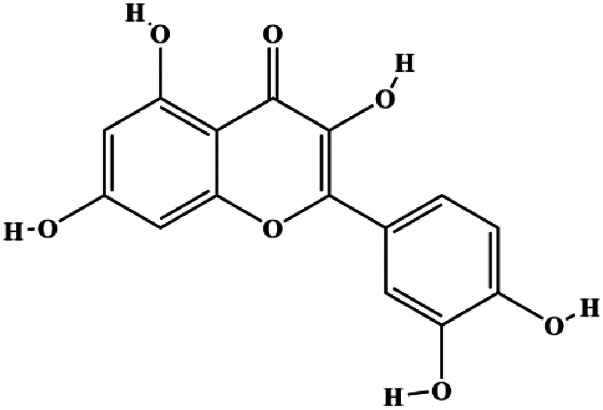	GAS5, Bax, caspase-3	Notch1, Jagged1, Hes1, Bcl-2	MCF-7	Jiang et al. (2021)
Rhamnetin (15,FPHAR_fphar-2021-801662_gs_fx121 20, 25 µM)	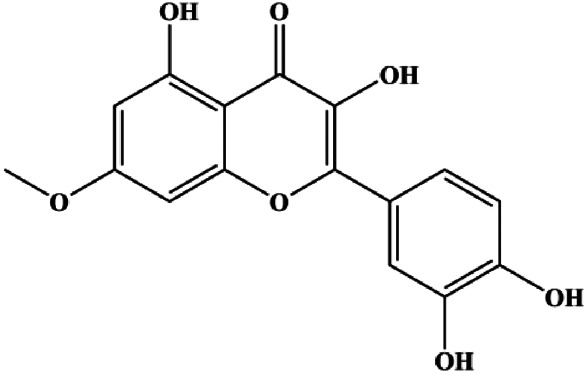	Activated caspase-3, activated caspase-9, (miR-)34a, p53	Notch1	MCF-7	Lan et al. (2019)
Shikonin (0.625, 1.25, 2.5 µM)	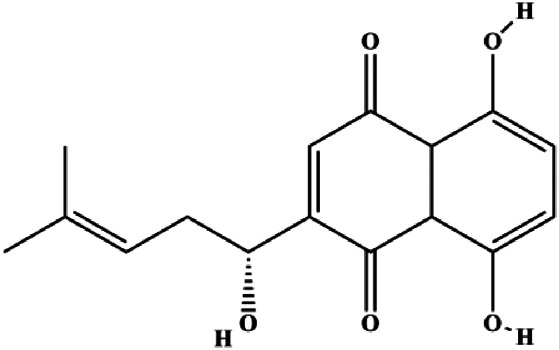		cIAP1, cIAP2, RIP1	MDA-MB-231	Wang et al. (2021)
Cordycepin (40, 80, 120 µM)	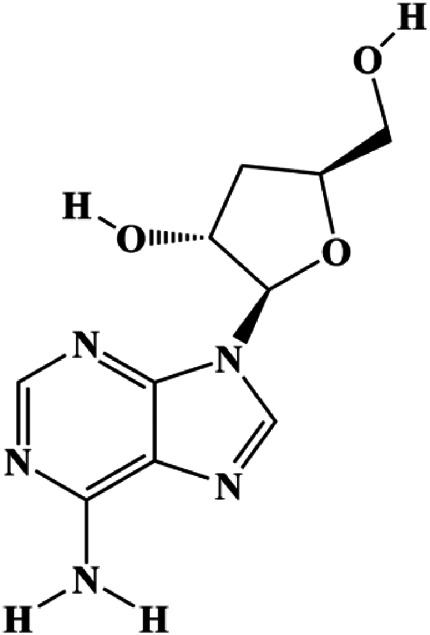	PUMA, CytoC, Fas, DR4/5, cleaved caspase-3	Bcl-2, XIAP, PDGFR-α	MDA-MB-231 MDA-MB-468 MCF-7	Liu et al. (2020)

### Mitochondrial Pathway–Mediated Apoptosis

The mitochondrial pathway can be triggered by various cellular stresses, such as UV radiation, DNA damage, ROS, hypoxia, and endoplasmic reticulum stress (ERS). It is also known as an intrinsic pathway that induces apoptosis. The mechanism of mitochondrial pathway–mediated apoptosis is related to altered mitochondrial membrane potential (MMP) and promotes cytochrome c (CytoC) release to activate initiator caspase-9, which then activated caspase-9 cleaves caspase-3, resulting in cell apoptosis. CytoC is regulated by the evolutionarily conserved B-cell lymphoma-2 (Bcl-2) family, which includes proapoptotic and antiapoptotic members (Suraweera et al., 2020). Antiapoptotic members, Bcl-2, Bcl-2–like (Bcl2L), and Bcl-2–related protein long isoform (BclXL), are located in the outer membrane of mitochondria and can prevent the release of CytoC. Proapoptotic members, Bcl-2–associated X-protein (Bax), BH3-interacting death domain (Bid), and Bcl-2–interacting protein (Bim), must be transferred to mitochondria to induce apoptosis.

**FIGURE 1:**
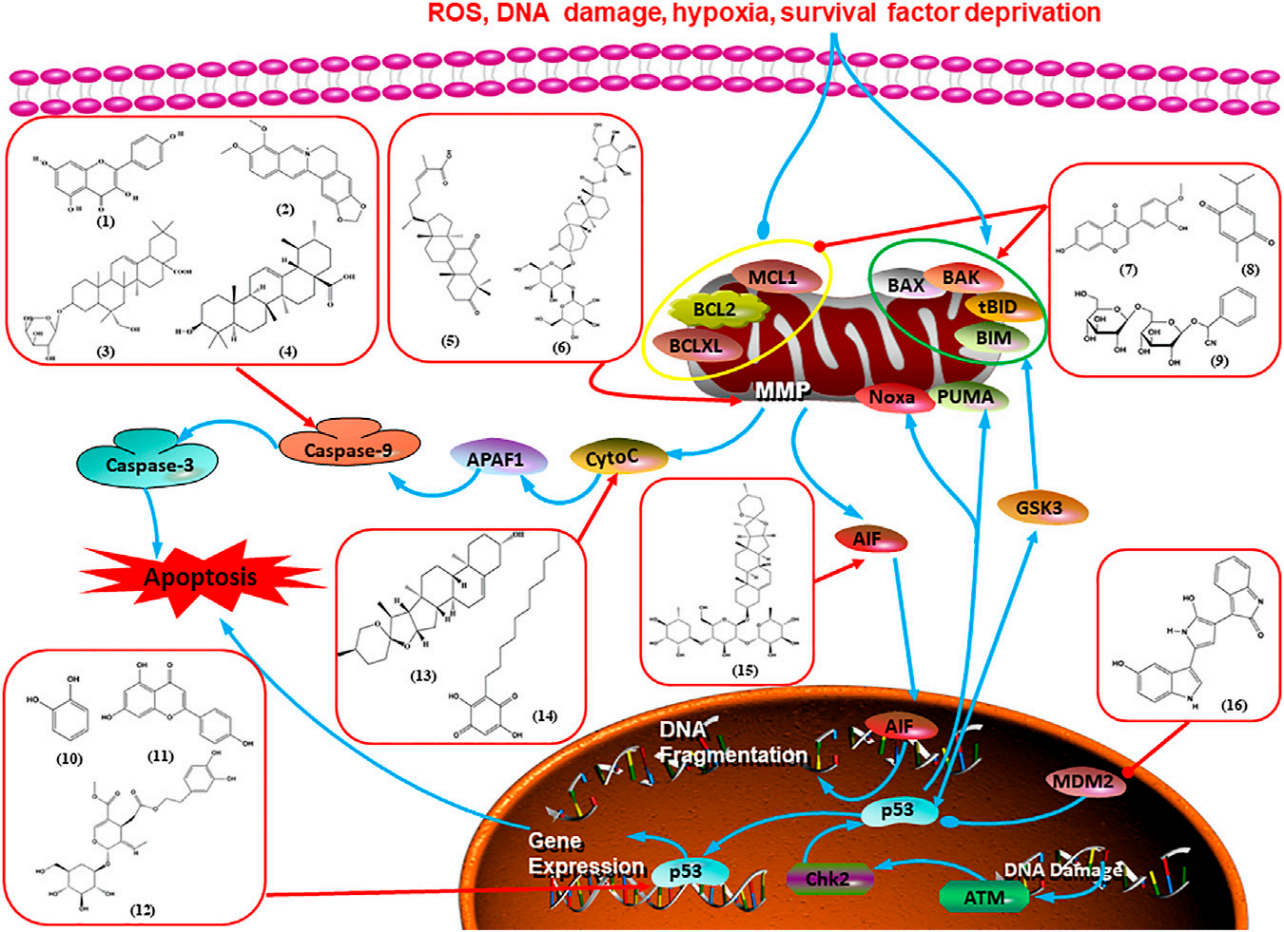
Natural products induced apoptosis through mitochondrial pathway. (1) Kaempferol, (2) berberine, (3) *Clematis* hederagenin saponin, (4) ursolic acid, (5) ganoderic acid DM, (6) stevioside, (7) Calycosin, (8) thymoquinon, (9) amygdalin, (10) catechol, (11) apigenin, (12) oleuropein, (13) diosgenin, (14) embelin, (15) dioscin, (16) violacein.

Numerous studies have suggested that certain natural products induce BC cell apoptosis through the mitochondrial pathway (Figure 1). Ganoderic acid DM is an active monomer of *Ganoderma lucidum*. In 2012, Wu et al. (2012) reported that ganoderic acid DM significantly induced DNA breakage and poly-ADP-ribose polymerase (PARP) cleavage, reduced MMP, and ultimately induced MCF-7 cell apoptosis. Sharma et al. (2012) also reported that 18β-glycyrrhetinic acid induced apoptosis through the mitochondrial death cascade, characterized by the absence of MMP, promoting the release of CytoC and activation of caspase-9. Stevioside is a diterpenoid glycoside found in *Stevia rebaudiana* (Bertoni) Bertoni leaves. A study by Paul et al. (2012) revealed that stevioside can reduce MMP and activate the mitochondrial-mediated apoptotic pathway in MCF-7 cells, suggesting that stevioside is a potent inducer of apoptosis. Amentoflavone, isolated from an ethyl acetate extract of *Selaginella tamariscina* (P. Beauv.) Spring, has been reported to trigger apoptosis of MCF-7 cells by reducing MMP and promoting CytoC release and caspase-3 activation (Pei et al., 2012). In 2013, as shown in the study by Tian et al. (2013), calycosin decreased Bcl-2 and increased Bax expression to incur BC cell apoptosis through the mitochondrial pathway. Rajput et al. (2013) found that thymoquinone, the predominant bioactive ingredient of black seed oil (*Nigella sativa* L.), could induce apoptosis in MDA-MB-468 and T47D cells through upregulation of the levels of Bax, CytoC, cleaved caspase-3, and cleaved PARP, along with downregulation of the levels of Bcl-2, BclXL, and survivin. Embelin is a small-molecule compound extracted from the Myrsinaceae family. As shown in the study by Li et al. (2013), embelin can regulate Bax and Bcl-2, promote the release of CytoC, and activate caspase-3 and caspase-9, which indicates that the mechanism of embelin-induced MCF-7 apoptosis is related to the mitochondrial pathway. In 2013, Kim et al. reported that ginseng saponin rg3 induced MDA-MB-231 cell apoptosis through typical mitochondria-dependent caspase activation (Kim et al., 2013). In 2014, Ma et al. reported that ursolic acid reduced the proliferation and induced apoptosis of MDA-MB-231 cells by increasing the activities of caspase-3 and caspase-9 via the mitochondrial apoptosis pathway (Ma and Sun, 2014). Kim et al. proposed that dioscin induced apoptosis in MDA-MB-231, MDA-MB-453, and T47D cells by promoting the transfer of apoptosis-inducing factor (AIF) from the mitochondria to the nucleus and inhibiting antiapoptotic proteins Bcl-2, cIAP-1, and Mcl-1 (Kim et al., 2014). As shown in the study by Zhang et al. (2018a), 20(S)-protopanaxadiol (PDD) upregulated the expression of Bax/Bcl-2, cleaved PARP, and downregulated the expression of MMP of MCF-7 cells, and the caspase protein family was activated, indicating that MCF-7 cell apoptosis was induced by PDD *via* the mitochondrial pathway. Paratocarpin E, which belongs to prenylated chalcone, was extracted from *Euphorbia humifusa* Wild. In 2016, Gao et al. (2016) reported that paratocarpin E induced apoptosis in MCF-7 cells by altering the expression of Bax and Bcl-2 and inducing the release of CytoC from the mitochondria into the cytoplasm, suggesting that mitochondria-mediated pathways are activated during this process. In 2016, Lee et al. reported that amygdalin induced apoptosis of Hs578T TNBC cells, and the proapoptotic effect may be related to Bcl-2 downregulation, Bax upregulation, caspase-3 activation, and cleaved PARP increase (Lee and Moon, 2016). Cordycepin, a major compound isolated from *Cordyceps sinensis* (BerK.) Sacc., has been reported to increase the activation of proapoptotic proteins, such as caspase-8, caspase-9, caspase-3, and Bax, and inhibit Bcl-2 expression, suggesting that cordycepin could induce apoptosis in BC cells via the caspase-dependent pathway (Wang et al., 2016). Berberine is an isoquinoline alkaloid isolated from *Cotridis rhizoma*. In 2017, Zhao et al. (2017) demonstrated that berberine activated caspase-9/CytoC–mediated apoptosis to suppress TNBC cell proliferation *in vitro* and *in vivo*. *Clematis* hederagenin saponin (CHS), isolated from *Clematis chinensis* Osbeck, is a triterpenoid saponin family. Cheng et al. (2018) reported that CHS could significantly increase mitochondrial Apaf-1 and CytoC to activate caspase-3 and caspase-9 in BC cells and finally showed a proapoptotic effect. In 2019, Zhu et al. reported that kaempferol, a flavonoid, could induce apoptosis in MDA-MB-231 cells, which may be achieved by increasing the expression of cleaved caspase-9/3 and p-ATM (Zhu and Xue, 2019). Liao et al. (2019) demonstrated that diosgenin induced the loss of MMP, resulting in the release of CytoC and activation of the caspase signaling cascade in BC cells. In a 2020 study by Rosas Gonzalez et al.(2020), allicin reduced cell viability and led to apoptosis by activating caspase-3/8/9; upregulating NOXA, p21, and Bak; and downregulating BclXL expression. Ma et al. (2021) found that dehydrocostuslactone suppressed the growth and promoted apoptosis in SK-BR-3 BC cells, which may be associated with the inhibition of the antiapoptotic ability of BC cells by regulating Bax/Bcl-2 and caspase-3 expression.

Cofilin can regulate mitochondrial division by interacting with Drp1 at mitochondrial division sites, leading to mitochondrial damage and CytoC release and ultimately inducing apoptosis. 4-Methylthiobutyl isothiocyanate (erucin) is a compound isolated from cruciferous vegetables. In 2015, Li et al. (2015) showed that erucin induced mitochondrial division and apoptosis in human BC cells through mitochondrial translocation of cofilin, which was correlated with downregulated PARP and upregulated caspase-3 and caspase-9 cleavage. Li et al. (2017a) reported that polyphyllin I induced mitochondrial translocation of DRP1 by dephosphorylating the Ser637 site of DRP1, leading to mitochondrial division and CytoC release from mitochondria into the cytoplasm, ultimately leading to BC cell apoptosis. Arnidiol is a pentacyclic triterpenoid diol. In 2020, Hu et al. (2020) found that arnidiol induced apoptosis in human BC MDA-MB-231 cells *via* mitochondrial translocation of Drp1 and cofilin, which was correlated with reduced PARP expression and increased caspase-3 cleavage. Furthermore, in 2021, Shen et al. (2021) revealed that coadministration of cepharanthine and epirubicin markedly led to mitochondrial translocation of cofilin, thus inducing apoptosis in TNBC cells *via* the mitochondrial pathway.

P53 is a nuclear transcription factor that is negatively and positively regulated by MDM2 and ATM, respectively, and can regulate apoptosis-related gene expression, such as that of Bax and BclXL, to exhibit an anti-BC effect. P53 can also alter MMP and trigger CytoC release, ultimately inducing BC cell apoptosis via the mitochondrial pathway. The deficiency of p53 reduces the therapeutic effect and prognosis of BC. Therefore, accumulating evidence has shown that many natural products can increase or stabilize p53 to induce BC apoptosis. Liu et al. (2012) proposed that apigenin induced p53-dependent apoptosis in BC T47D cells. In 2014, Patel et al. revealed that l-carvone induced p53- and caspase-3–mediated apoptosis in MCF-7 and MDA-MB-231 cells (Patel and Thakkar, 2014). In 2014, Hassan et al. proposed that oleuropein-induced apoptosis via the p53 pathway in MCF-7 cells was related to the upregulation of p53 and Bax and downregulation of Bcl-2 (Hassan et al., 2014). In 2015, Zu et al. reported that emodin reduced Bcl-2 levels and increased cleaved caspase-3, PARP, p53, and Bax levels in BCAP-37 and ZR-75-30 BC cells, suggesting that it could induce apoptosis through the p53-mediated mitochondrial pathway (Zu et al., 2015). Gaillardin is a natural sesquiterpene lactone. In 2015, Fallahian et al. found that the mechanism of apoptosis induced by gaillardin in MCF-7 and MDA-MB-468 cells involved upregulation the expression of Bax and p53, increased the production of ROS, and downregulated the expression of Bcl-2, inducing the loss of MMP (Fallahian et al., 2015). Sesamin is a major component of *Sesamum indicum* L. In 2015, Siao et al. demonstrated that sesamin could cause apoptosis in BC cells MCF-7 by improving the expression of the apoptosis markers Bax, caspase-3, and p53 (Siao et al., 2015). Zhang et al. found that osthole induced apoptosis in MCF-7 cells by the p53 pathway, which was related to high expression of Bax, p53, p21, and CytoC, as well as downregulation of Bcl-2 and MMP (Zhang and Zhu, 2016). Feng et al. (2016) showed that high concentrations of arecoline resulted in MCF-7 cell apoptosis, which may be achieved by increasing the protein expression of p53 and Bax and decreasing that of Bcl-2. Violacein is a natural purple pigment produced primarily by microorganisms. Alshatwi et al. (2016) showed that violacein induced apoptosis in MCF-7 cells through the upregulation the levels of Bax and p53 and downregulation the levels of MDM2. Li et al. (2017b) demonstrated that the mechanism of apoptosis induced by liriodenine in MCF-7 cells inhibited the expression of Bcl-2, cyclin D1, and vascular endothelial growth factor (VEGF) and upregulated the level of p53. As shown in the study by Liang et al. (2021), resveratrol can facilitate TNBC cell apoptosis *via* the p53-mediated pathway. In 2021, a study by Vazhappilly et al. indicated that catechol could inhibit proliferation and result in apoptosis in MCF-7 cells by activating p53, inhibiting of regulatory proteins such as DNMT1, P-BRCA1, Mcl-1, and PDCD6, and increasing the Bax/Bcl-2 ratio. Ultimately, caspase-mediated cell death is triggered (Vazhappilly et al., 2021). In their study, Sp et al. (2021) showed that 6-zingerol might induce apoptosis in MDA-MB-231 and MCF-7 BC cell lines by activating p53, regulating the Bax/Bcl-2 ratio, and increasing the release of CytoC.

Mitochondria are the main sites of ROS production. Accumulated ROS can directly act on the mitochondrial membrane, induce changes in MMP, release CytoC and other AIFs, and finally activate the caspase cascade, leading to apoptosis. 3*β*, 6*β*, 16*β*-trihydroxylup-20 (29)-ene (TTHL) is a pentacyclic triterpene isolated from the medicinal plant *Combretum leprosum* Mart. In 2014, Viau et al. demonstrated that TTHL induced MCF-7 cell apoptosis, and TTHL-induced apoptosis was accompanied by increased caspase-9 and intracellular ROS (Viau et al., 2014). Baicalein is a natural flavonoid extracted from *Scutellaria baicalensis* Georgi. A study by Liu et al. (2019) showed that baicalein damaged MMP, downregulated antiapoptotic protein Bcl-2 expression, upregulated proapoptotic protein Bax expression, and promoted the release of CytoC and activated caspase-9/3 in BC cells by increasing intracellular ROS levels. In a study conducted by Wang et al. (2020), diosmetin significantly upregulated the protein expression of p53, Bax, and caspase-3; downregulated the protein expression of Bcl-2; reduced MMP; and promoted ROS accumulation in MCF-7 cells, suggesting that diosmetin may promote apoptosis in MCF-7 cells through ROS- and p53-mediated mitochondrial apoptosis pathways.

### FasL-Fas–Mediated Apoptosis

**FIGURE 2:**
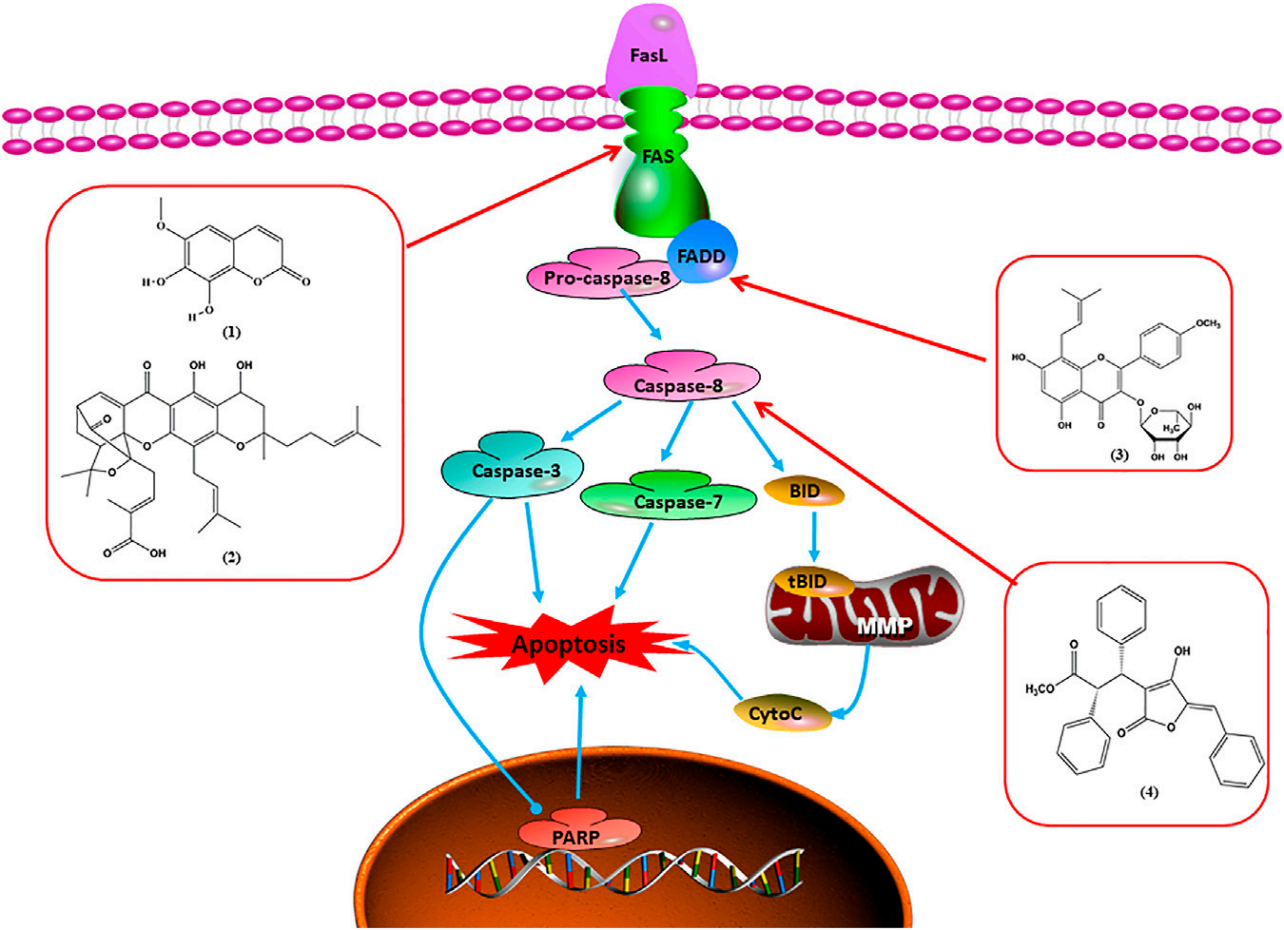
Natural products induced apoptosis through FasL-Fas pathway. (1) Fraxetin, (2) gambogenic acid, (3) icariside II, (4) pulveraven A.

Upon FasL binding to the Fas receptor, it causes the formation of the complex of Fas, FADD, and pro–caspase-8, known as the death-inducing signaling complex. After caspase-8 is activated through self-cleavage of pro–caspase-8, it subsequently activates downstream effectors, including caspase-3 and caspase-7, resulting in cell apoptosis. Therefore, targeting to the FasL-Fas pathway is one of the strategies to induce cell apoptosis. Certain natural products have been shown to activate the FasL-Fas pathway to induce apoptosis in BC cells (Figure 2).

In 2012, Huang et al. (2012) reported that icariside II enhanced the expression of Fas and FADD, activated caspase-8 in MCF-7 BC cells, and played a role in promoting apoptosis. Gambogenic acid is the main active component isolated from the tree *Garcinia hanburyi* Hook. f. In 2013, Zhou et al. (2013) proposed that gambogic acid could induce BC cell apoptosis, and the mechanism was also related to increased expression of Fas, cleaved caspase-3/8/9, and Bax and decreased expression of antiapoptotic protein Bcl-2. In 2013, Wang et al. (2013a) proposed that genistein enhanced FasL, FADD, and cleaved caspase-8 protein expression in MDA-MB-231 cells, suggesting that genistein-induced apoptosis was mediated by the Fas/FasL pathway. In 2014, Li et al. (2014) demonstrated that *α*-mangostin significantly inhibited the expression of Fas and intracellular Fas activity to induce apoptosis in BC cells. In 2017, Liu et al. (2017a) reported that fraxetin suppressed cell proliferation and induced MCF-7 cell apoptosis by increasing the expression of Fas, FasL, and Bax and reducing Bcl-2 expression. Pulveraven A is a phenolic compound isolated from *Pulveroboletus ravenelii*. In 2021, Lee et al. proposed that apoptotic BC death was induced by the activation of promoter caspase-8 and executioner caspase-7 and upregulated the expression of FADD by pulveraven A. Furthermore, it was accompanied by an increase in the Bax/Bcl-2 ratio (Lee et al., 2021).

### PI3K/AKT Pathway Mediated Apoptosis

**FIGURE 3:**
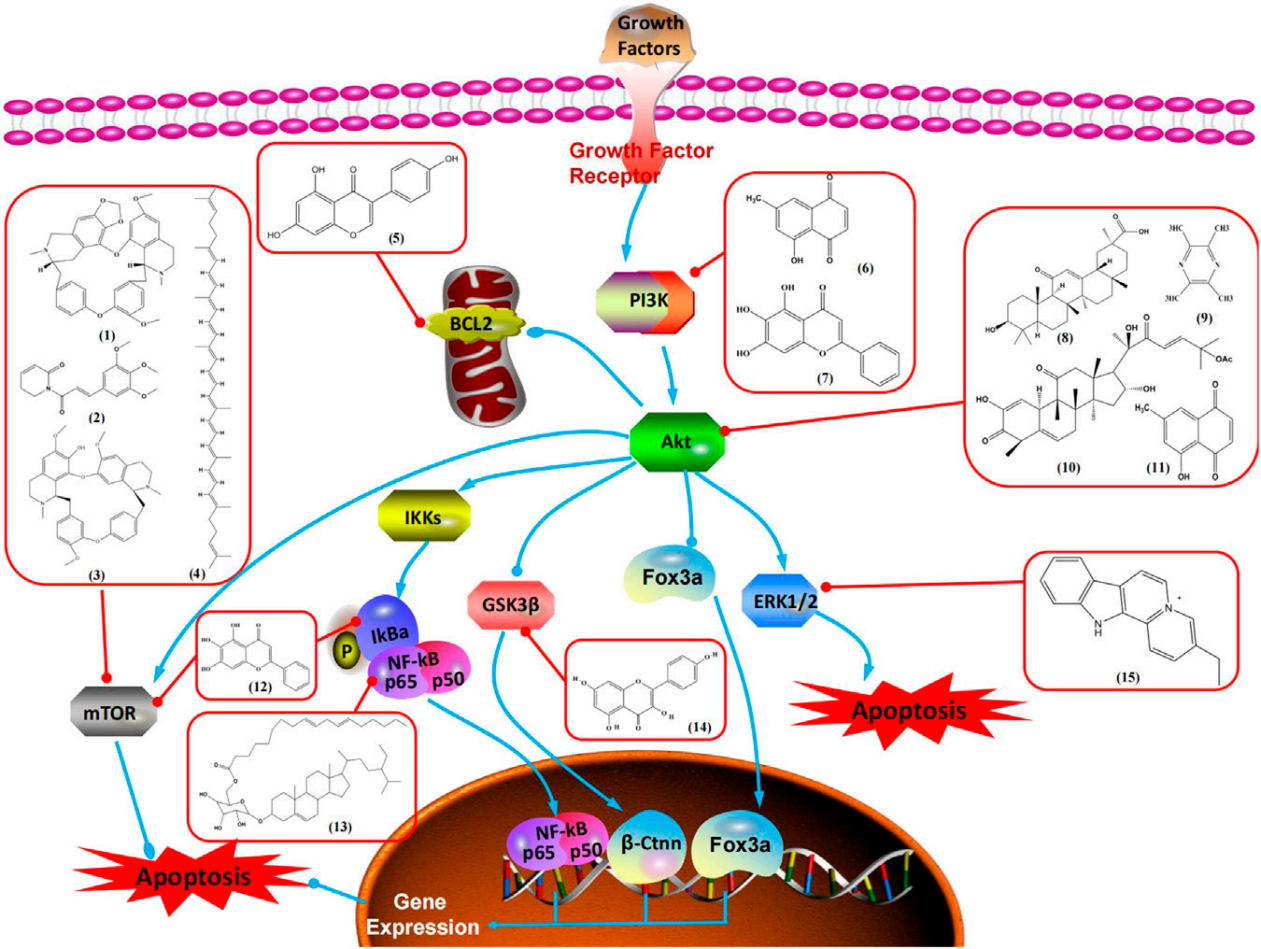
Natural products induced apoptosis through PI3K/AKT pathway. (1) cepharanthine, (2) piperlongumine, (3) fangchinoline, (4) lycopene, (5) genistein, (6) ramentaceone, (7) baicalein, (8) 18β-glycyrrhetinic acid, (9) tetramethylpyrazine, (10) cucurbitacin e, (11) ramentaceone, (12) baicalein, (13) daucosterol linoleate, (14) kaempferol, (15) flavopereirine.

The PI3K/AKT pathway is triggered by the binding of ligands, such as insulin, growth factors, and hormones. Upon PI3K activation, it phosphorylates AKT, and activated AKT plays an important role in regulating Bad, a proapoptotic member of the Bcl-2 family, to inhibit mitochondrial-mediated apoptosis. Alternatively, activated AKT phosphorylates other components, such as the mTOR complex and IkBα/NF-κB, which are ultimately involved in cell growth (Miricescu et al., 2020). Therefore, targeted inhibition of PI3K/AKT pathway activation can promote cell apoptosis. In recent decades, increasing evidence has indicated that nature produces can inactivate PI3K/AKT pathway to induce BC apoptosis (Figure 3).

In 2012, Sharma et al. (2012) reported that 18-β-glycyrrhetinic acid induced apoptosis by modulating the AKT/Foxo3a/Bim pathway in MCF-7 cells. In 2015, Hu et al. (2015) showed that isorhamnetin had a proapoptotic effect in MCF-7 and MDA-MB-468 BC cells, which was mediated by the AKT and MEK signaling pathways. In 2015, another report by Chen et al. (2015c) suggested that the phosphorylation level of AKT and the expression of its downstream target protein HOTAIR were decreased after treatment with calycosin, thus inducing apoptosis in MCF-7 BC cells. In 2016, Peng et al. (2016) proposed that ginsenoside Rg3 promoted apoptosis in BC cells by interfering with the level of mammaglobin A in MDA-MB-231 cells, and this effect was achieved by affecting PI3K/AKT signaling pathway activity. *α*-Mangostin was extracted from mangosteen (*Garcinia mangostana* L.). In 2016, Kritsanawong et al. (2016) found that α-mangostin induced apoptosis in BC cells, which may be associated with PI3K/AKT signaling pathway regulation. Stachydrine hydrochloride is a well-known bioactive ingredient extracted from *Leonurus cardiac* L. Wang et al. (2017) reported that stachydrine hydrochloride inhibited cell division and growth and induced apoptosis in BC cells *via* deactivation of the AKT and ERK pathways. As a rare ginsenoside and the main ingredient extracted from fine black *Panax ginseng* C.A.Mey., ginsenoside Rg5, can induce apoptosis in a dose-dependent manner by inhibiting p-PI3K and p-AKT *in vivo* (Liu and Fan, 2018). In 2018, Liu et al. (2018b) reported that galangin inhibited MCF-7 cell proliferation and induced cell apoptosis *via* the mitochondrial and PI3K/AKT pathways. In 2018, Shen et al. (2018) showed that tetramethylpyrazine could significantly decrease the gene expression and the activity of AKT and increase the activity of caspase-3, thus inducing apoptosis in BC cells. Daucosterol linoleate, a steroid extracted from *Manihot esculenta* Crantz, has been reported to diminish the expression of the antiapoptotic proteins BclXL, Bcl-2, and XIAP; promote the levels of proapoptotic proteins Bax and Bad; and activate caspase-dependent apoptosis *in vivo*. Moreover, daucosterol linoleate invalidates the upstream of the PI3k/AKT/NF-κB pathway (Han et al., 2018). Crambescidin, separated from *Monanchora viridis*, a marine sponge, may be involved in the inactivation of phosphorylation of Akt, NF-κB, and MAPK pathways, resulting in apoptosis in TNBC cells according to the study by Shrestha et al. (2018). In 2019, Zhao et al. (2019) showed that kaempferol promoted the apoptosis of the SUM190 inflammatory BC cell line, and the proapoptotic effect might be associated with PI3K/AKT/GSK-3β pathway inhibition. Flavopereirine is a β-carboline alkaloid with antiplasmodial activity. In 2019, Yeh et al. (2019) reported that flavopereirine can induce apoptosis in MDA-MB-231cells by inhibiting the AKT/p38/ERK pathway. In 2021, Xu et al. (2021) reported that erianin can induce TNBC cell apoptosis, which may be ascribed to the inhibition of the PI3K/AKT pathway.

mTOR is one of the downstream members of the PI3K/AKT signaling pathway and is directly regulated by AKT. Activation of mTOR can phosphorylate p70S6K and 4EBP1 to promote cell survival. In 2017, Zhang et al. discovered that fangchinoline induced apoptosis in MDA-MB-231 cells. The mechanism was related to the downregulation of PI3K, AKT, mTOR, and p-PI3K, p-AKT, and p-mTOR proteins in a concentration-dependent manner (Zhang et al., 2017). In 2014, Takeshima et al. (2014) proposed that lycopene induced apoptosis in the MDA-MB-468 cell line by inhibiting the phosphorylation of AKT and its downstream molecule mTOR, enhancing PARP cleavage and upregulating Bax. In 2014, Shrivastava et al. proposed that piperlongumine, an alkaloid, reduced the expression of p-AKT, p70S6K1, 4EBP1, Bcl-2, and p53 and increased the expression of Bax and CytoC in human TNBC cells, suggesting that the proapoptotic function of piperlongumine was related to the inhibition of the PI3K/AKT/mTOR signaling axis (Shrivastava et al., 2014). Cepharanthine is a biscoclaurine alkaloid extracted from *Stephania cephalantha* Hayata. In 2017, Gao et al. (2017) reported that cepharanthine resulted in apoptosis in MCF-7 and MDA-MB-231 cells, which might be achieved by AKT/mTOR signaling pathway inhibition. In 2017, Sun et al. proposed that fisetin reduced the phosphorylation of PI3K, AKT, mTOR, and p70S6K and upregulated apoptosis factors such as Bax and caspase-3/8/9 in BC cells. These results indicated that fisetin inhibited cell growth and induced the apoptosis, which was attributed to the inhibition of the PI3K/AKT/mTOR signaling pathway (Sun et al., 2018). *Paris* saponins are saponin compounds isolated from *Paris polyphylla* Sm. A study by Xie et al. showed that the apoptotic effect of *Paris* saponins in BC cells was mediated through the Akt/mTOR signaling pathway. It was highly correlated with the downregulation of p-AKT, p-mTOR, p-P70S6K, and p-4EBP1 (Xie et al., 2017). According to another study by Yan et al., baicalein significantly reduced the expression of p-AKT, p-mTOR, NF-κB, and p-IκB in MCF-7 and MDA-MB-231 cells, demonstrating that baicalein can induce BC cell line apoptosis in animal models by inhibiting the PI3K/AKT signaling pathway (Yan et al., 2018). 20(S)-PPD belongs to one of the main active metabolites of *Panax ginseng* C.A.Mey. In 2018, Zhang et al. (2018a) reported that 20(S)-PPD could downregulate the protein expression of PI3K/AKT/mTOR in MCF-7 cells. In 2018, Sultan et al. (2018) reported that cannabidiol suppressed cell survival and prompted apoptosis in a dose-dependent manner, accompanied by downregulation of mTOR and upregulation and localization of PPARγ protein in the nuclei and cytoplasm. In 2018, another report by Hu et al. (2018) indicated that curcumin inhibited the phosphorylation of AKT and mTOR, decreased Bcl-2 and increased Bax, and cleaved caspase-3, subsequently inducing apoptosis in MCF-7 and MDA-MB-231 cells. In 2020, Wei et al. (2020) proposed that magnoflorine, a quaternary alkaloid separated from *Schisandra chinensis* (Turcz.) Baill or *Aristolochia clematitis* L., improved cell sensitivity to doxorubicin by inducing apoptosis through the Akt/mTOR and p38 signaling pathways.

Bcl-2 family proteins, which play a key role in determining cell death or survival, are downstream targets of AKT. For example, AKT directly phosphorylates Bad, which is then separated from Bcl-2. Then, Bcl-2 binds to the proapoptotic Bcl-2 family members such as Bax and Bak, thereby inhibiting apoptosis. In 2014, Kong et al. (2014) reported that cucurbitacin E can induce the MDA-MB-468 cell apoptosis by reducing the levels of cyclin D1, survivin, XIAP, Bcl-2, and Mcl-1 and inhibiting the activation of AKT. d-Rhamnose *β* hederin is an oleanane-type triterpenoid soap, which was found to induce apoptosis in BC cells by inhibiting the PI3K/AKT signaling pathway and regulating the protein expression of the Bcl-2 family (Cheng et al., 2014). Genistein, an estrogenic soy-derived compound, belongs to the isoflavone family. In 2015, Chen et al. (2015b) proposed that genistein induced apoptosis via the IGF-1R/p-Akt signaling pathway in MCF-7 cells, accompanied by a reduction in the ratio of Bcl-2/Bax. Ramentaceone, a naphthoquinone isolated from *Drosera rotundifolia* L., was reported to induce BC cell apoptosis by inhibiting the PI3K/AKT signaling pathway, which was highly correlated with upregulated Bax and Bak expression, downregulated Bcl-2 expression, and inhibition of PI3K and AKT phosphorylation (Kawiak and Lojkowska, 2016). A study by Razak et al. (2019) showed that eupatorine-induced apoptosis in MCF-7 and MDA-MB-231 cells was mediated by the upregulation of proapoptotic genes such as Bak1, HIF1A, Bax, and Bad, increasing CytoC release and SMAC/Diablo and blocking the p-AKT pathway. Hong et al. showed that ginsenoside Rk1 increased the levels of Bax, CytoC, and cleaved caspase-3/8/9 and reduced Bcl-2 by blocking the PI3K/AKT pathway (Hong and Fan, 2019). In 2019, Han et al. (2019) showed that parthenolide could lead to BC cell apoptosis by modulating the PI3K/AKT signaling pathway, which was highly correlated with downregulated Bcl-2 expression and upregulated cleaved caspase-3 expression. In 2020, Yu et al. proposed that avicularin induces MDA-MB-231 cell apoptosis, which may be associated with the inhibition of the PI3K/AKT signaling pathway to decrease the gene expression of Bcl-2, BclXL, and CDK2 (Yu et al., 2020).

### MAPK Pathway–Mediated Apoptosis

MAPK pathways are sequentially phosphorylated and activated by three protein kinases: MAPKKK activates MAPKK, which then activates MAPK. MAPKs are traditionally classified as ERK, JNK, and p38 kinase in mammalian cells. Through cascade reactions, MAPKs can transmit extracellular and intracellular signals to regulate cell growth, proliferation, differentiation, migration, and apoptosis.

**FIGURE 4:**
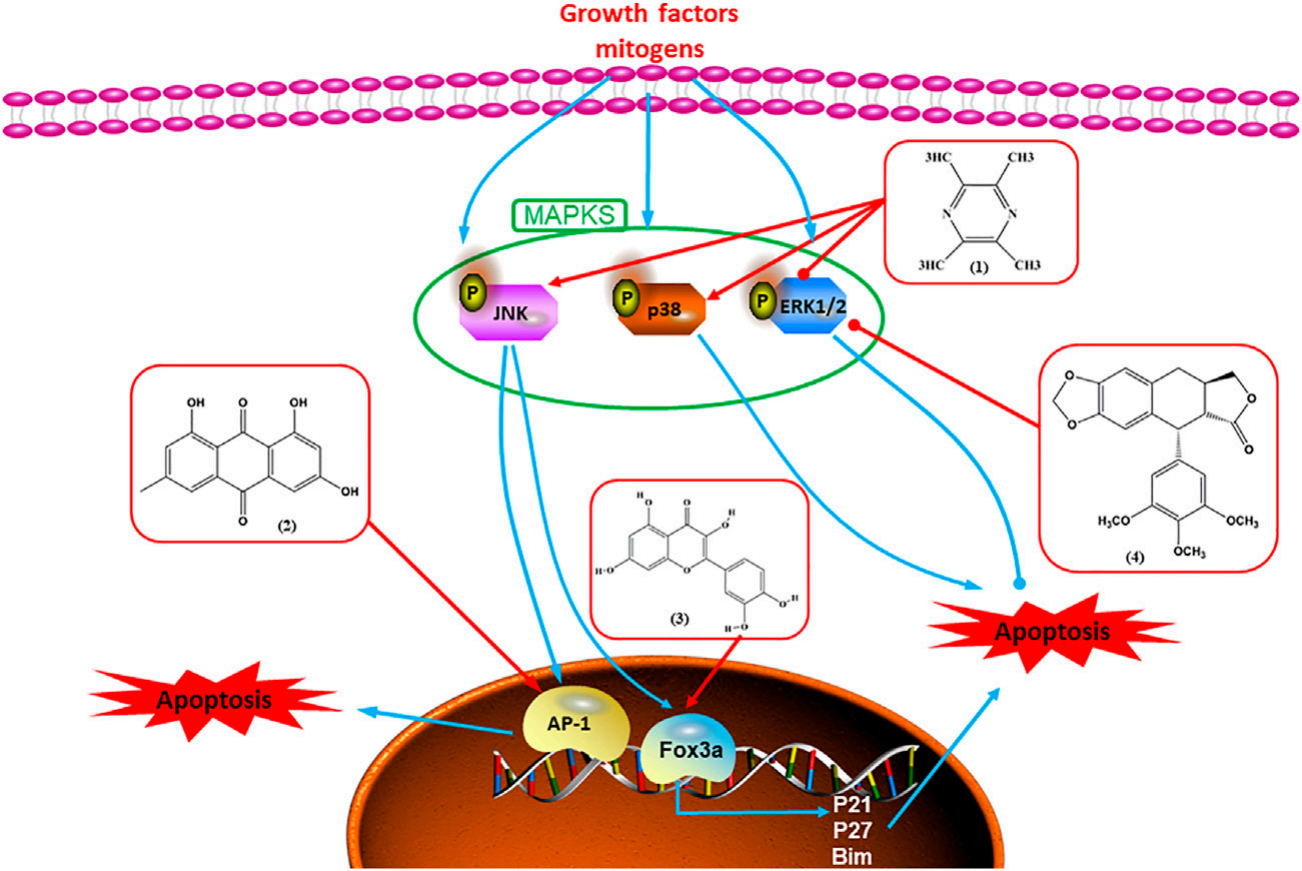
Natural products induced apoptosis through MAPK pathway. (1) Tetramethylpyrazine, (2) emodin, (3) quercetin, (4) Deoxypodophyllotoxin.

Accumulating evidence suggests that MAPKs are important targets in natural product–induced BC cell apoptosis (Figure 4). In 2012, Chen and Sun found that formononetin elevated the Bax/Bcl-2 ratio in MCF-7 cells by activating the Ras-p38MAPK signaling pathway and finally induced cell apoptosis (Chen and Sun, 2012). Kim et al. reported that genipin, a constituent of *Gardenia jasminoides* J. Ellis, downregulated Bcl-2 and upregulated Bax and cleaved caspase-3 by activation of p38 and JNK signals, suggesting that genipin induced MDA-MB-231 cell apoptosis mediated by MAPK pathway activation (Kim et al., 2012). Deoxypodophyllotoxin (DTP) is a natural ingredient extracted from *Juniperus communis* L. In 2015, Benzina et al. proposed that DTP can induce BC cell apoptosis by inactivation of the ERK and NF-κB signaling pathways (Benzina et al., 2015). In 2015, Fan et al. (2015) proposed that emodin suppressed MCF-7 cell proliferation, and further studies showed that emodin promoted MCF-7 cell apoptosis by activating the JNK-AP1 signal transduction pathway. In 2015, Ranganathan et al. (2015) demonstrated that quercetin significantly inhibited cyclin D1, p21, Twist, and p-p38 expression in MCF-7 cells, demonstrating that quercetin increased BC cell apoptosis through the p38-Twist pathway mediated by cell cycle arrest. In 2017, Nguyen et al. also found that quercetin increased the signaling activities of p53, p21, and GADD45, and Foxo3a protein and mRNA expression and induced nuclear translocation of Foxo3a. The JNK inhibitor abolished quercetin-stimulated Foxo3a activity and apoptosis, suggesting that quercetin induced apoptosis by regulating Foxo3a signaling in TNBC cells (Nguyen et al., 2017). Isocryptotanshinone is a natural bioactive component isolated from *Salvia miltiorrhiza* Bunge. In 2015, Zhang et al. reported that isocryptotanshinone promoted apoptosis in human MCF-7 BC cells by activating MAPK signaling pathways, including p38, ERK, and JNK (Zhang et al., 2015a). In 2016, as shown in the study by Lai et al., tetramethylpyrazine (TMP) improved the expression of p-p38 and p-JNK proteins. However, it reduced p-ERK expression in MCF-7 cells. TMP can induce apoptosis in MCF-7 cells, and this may be related to MAPK signaling pathway activation (Lai et al., 2016). α-Mangostin was extracted from *Garcinia mangostana* L*.*, and in 2016, Kritsanawong et al. (2016) found that α-mangostin could promote BC cell apoptosis by inhibiting PI3K/AKT and increasing p38 and JNK activation. In 2017, Lee et al. found that gallic acid decreased the expression of cyclin D1/CDK4 and cyclin E/CDK2, increased the expression of p21 and p27, and induced the activation of caspase-3/9 in MDA-MB-231 cells. In addition, p38 was found to be involved in gallic acid–induced apoptosis. The results indicated that gallic acid–induced apoptosis was related to the p38/p21/p27 axis (Lee et al., 2017). In 2019, Huang et al. (2019) reported that sophoraflavanone G suppressed the MAPK-related pathway to increase cleaved caspase-3/8/9 and Bax expression, decrease Bcl-2 and BclXL expression, and prompt the release of CytoC from mitochondria to the cytoplasm in MDA-MB-231 cells. Cardamonin is natural chalcone separated from large black *Elettaria cardamomum* (L.) Maton. As shown in the study by Kong et al. (2019), cardamonin increased the expression of Foxo3a and its target genes p21, p27, and Bim, and further studies suggested that cardamonin induced BC cell apoptosis by activating the JNK-Foxo3a pathway. In 2021, another report by Kim et al. (2021) indicated that silymarin induced BC cell apoptosis by regulating the MAPK signaling pathway, which increased the levels of Bax, cleaved PARP, cleaved caspase-9, and p-JNK and reduced the levels of Bcl-2, p-p38, and p-ERK.

### NF-κB–Mediated Apoptosis

NF-κB can be activated physiologically and pathologically, such as by growth factors and oxidant-free radicals, particularly by pro-inflammatory cytokines interleukin 1 (IL-1) and tumor necrosis factor (TNF). Binding to the receptor TNFR1, TNF can recruit TNF-associated receptor death domain that binds to the TNF receptor–associated factor 2 and the kinase receptor–interacting protein 1, and then recruit IκB kinase (IKK), leading to the phosphorylation and subsequent degradation of IκBα, which activates NF-κB translocation into the nucleus, thereby regulating related gene expression to regulate cell apoptosis.

In 2013, Wang et al. (2013b) demonstrated that oridonin induced MDA-MB-231 BC cell apoptosis, which was associated with a reduction in the Bcl-2/Bax ratio, caspase-8, NF-κBp65, IKKα, IKKβ, and p-mTOR, and upregulated cleaved PARP, PPAR, and Fas expression levels. In 2018, Wu et al. (2018b) proposed that delphintin significantly downregulated the expression of p-NF-κBp65, p-IκBα, p-IKKα/β, and p-PKCα in MDA-MB-453 and BT-474 BC cells. The results showed that delphintin induced apoptosis by blocking the NF-κB signaling pathway. In 2018, Ren et al. demonstrated that chrysophanol upregulated caspase-3 and PARP cleavage and downregulated the apoptosis regulators Bcl-2, p-NF-κBp65, and p-IκB, which indicated that the proapoptotic function of chrysophanol was achieved by regulating the NF-κB/Bcl-2 signaling cascade (Ren et al., 2018). In 2021, Chen et al. found that capsaicin significantly reduced the expression of FBI-1, Ki-67, Bcl-2, and survivin; increased Bax protein expression; and activated caspase-3 in BC cells. Furthermore, NF-κB, the target gene of FBI-1, was also inactivated by capsaicin treatment. These results indicate that the proapoptotic effect of capsaicin is related to the FBI-1–mediated NF-κB pathway (Chen et al., 2021).

### ROS-Mediated Apoptosis

**FIGURE 5:**
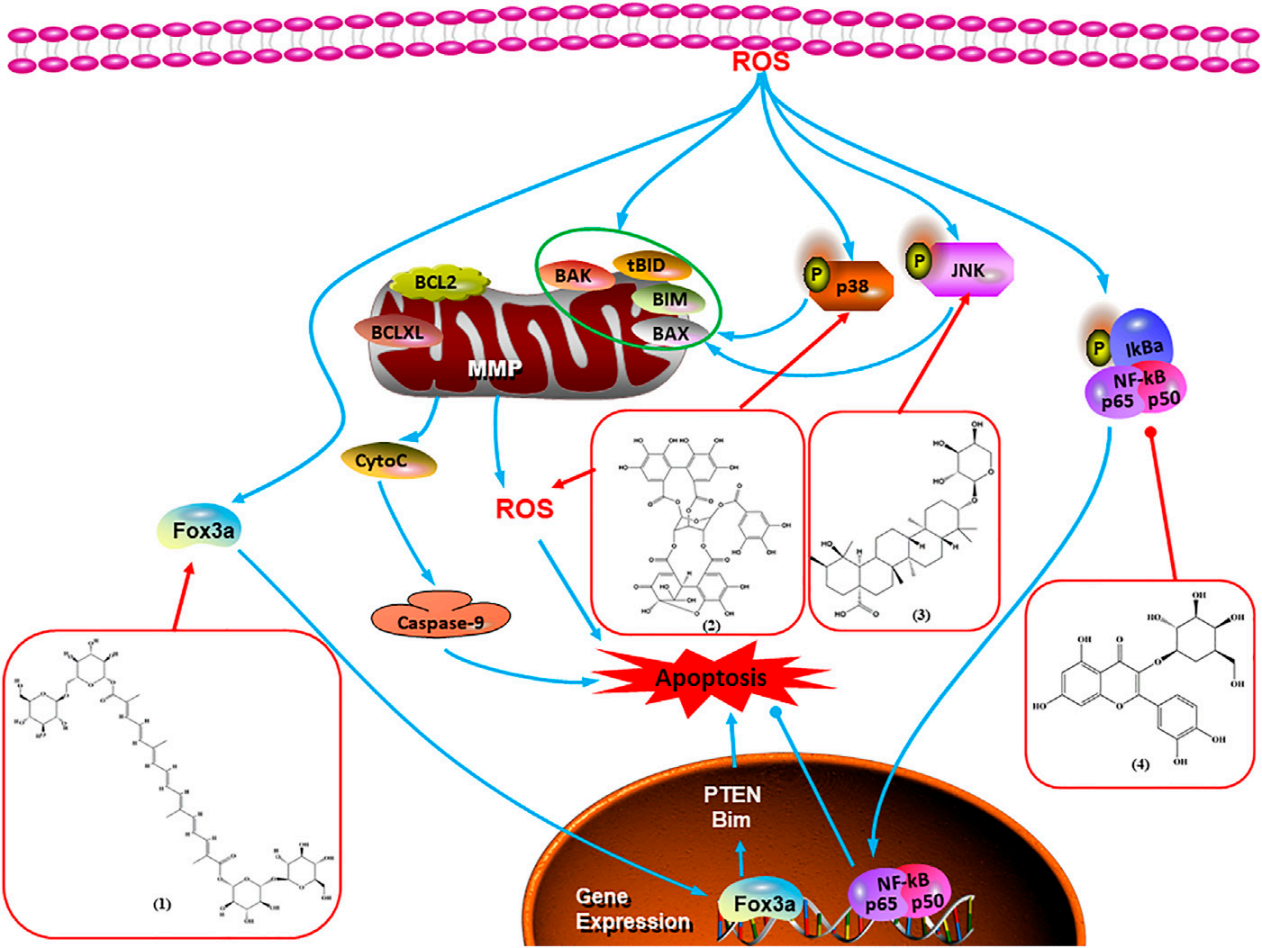
Natural products induced apoptosis through ROS pathway. (1) Crocin, (2) geraniin, (3) ziyuglycoside II, (4) hyperoside.

Intracellular ROS can induce apoptosis through several signal pathways. First, ROS can cause oxidative damage to all mitochondrial components. Damaged mitochondrial DNA disrupts mitochondrial oxidative phosphorylation, thus increasing MMP and releasing CytoC to contribute to cell death. In addition, ROS can activate JNK and p38 and trigger mitochondria-mediated apoptosis. Moreover, Foxo3a is a nuclear transcription factor, and ROS can promote the translocation of Foxo3a, which regulates apoptosis-related gene expression. However, ROS can increase NF-κB activation and inhibit apoptosis. Hence, ROS-mediated apoptosis may increase with the use of natural components (Figure 5).

In 2014, a study showed that ziyuglycoside II treatment induced BC cell apoptosis by activating the ROS/JNK pathway (Zhu et al., 2014). In 2015, Xie et al. proposed that berberine also increased the production of ROS in MCF-7 and MDA-MB-231 cells, which promoted proapoptotic JNK signaling. Phosphorylated JNK triggers MMP depolarization and Bcl-2 downregulation, concomitant with the Bax upregulation, and release of CytoC and AIF from mitochondria, eventually leading to apoptosis (Xie et al., 2015). Geraniin, a typical ellagitannin extracted from *Phyllanthus urinaria* L., was found to contain a series of bioactive. In 2016, Zhai et al. (2016) reported that geraniin could generate intracellular ROS and activate p38, and inhibition of ROS can restrain the phosphorylation of p38 and reverse geraniin-induced apoptosis, which suggests that geraniin results in MCF-7 cell apoptosis via the ROS–p38 pathway. In 2019, Qiu et al. (2019) proposed that hyperoside induced apoptosis in MCF-7 and 4T1 cells through the ROS-mediated NF-κB signaling pathway, which was attributed to reduced levels of Bcl-2 and XIAP, and increased the expression of Bax and cleaved caspase-3. In 2020, Nasimian et al. found that crocin induced apoptosis in MCF-7 and MDA-MB-231 cells. The ROS-activated Foxo3a cascade played a crucial role in this process, and Foxo3a-mediated upregulation of PTEN further inhibited the AKT pathway (Nasimian et al., 2020).

Mitochondria are the primary site of ROS production and are also one of the most sensitive targets of ROS action. Intracellular ROS accumulation can activate the mitochondria-mediated apoptosis pathway. Fucoidan is an active ingredient in seaweeds. In 2013, according to the study by Banafa et al. (2013), fucoidan induced MCF-7 cell apoptosis by upregulating caspase-8 and Bax, downregulating Bcl-2, and increasing the release of CytoC and the production of ROS. Looi et al. also reported in 2013 that vernodalin can induce MCF-7 cell apoptosis by increasing the production of ROS in human BC cells, thereby inducing the reduction of MMP and the release of CytoC, thus triggering the caspase cascade and PARP cleavage (Looi et al., 2013). Tetrandrine is an alkaloid that is known for its anticancer activity. In 2020, N et al. (2019) demonstrated that tetrandrine separated from *Cyclea peltata* (Lam.) Hook. f. and Thomson induced cytotoxicity and apoptosis by increasing ROS and caspase-8/9/3 in MDA-MB-231 cells. Pereyra-Vergara et al. (2020) reported that apoptosis induced by (−)-epicatechin in human BC cells was mediated by ROS to increase the proapoptotic proteins Bad and Bax. In 2020, Jia et al. (2020) proposed that formononetin could induce mitochondrial damage and decrease MMP in BC cells by inhibiting antioxidant enzyme activity and increasing ROS levels, ultimately activating caspase-3-mediated apoptosis.

### JAK-STAT3–Mediated Apoptosis

JAK/STAT signaling can transfer an extracellular signal into a transcriptional response that contributes to cancer progression and metastatic development. IL-6, epidermal growth factor (EGF), interferon-α, and other extracellular signals can stimulate the activation of JAK2 and STAT3. Phosphorylated STAT3 then enters the nucleus; dimerized STAT3 binding to specific regulatory sequences could activate or inhibit transcription of target genes, such as BclXL, p21, and Myc, to regulate cell apoptosis.

For natural products, inhibiting the JAK/STAT3 pathway may be a potential strategy to induce BC cell apoptosis. In 2014, Xu et al. reported that apigenin can induce MDA-MB-453 cell apoptosis by upregulating cleaved caspase-8/3 and PARP, blocking the activation (phosphorylation) of JAK2 and STAT3, and decreasing the nuclear tillering of STAT3 (Seo et al., 2014). In 2015, Zhang et al. demonstrated that berberine upregulated Bax, cleaved PARP, and caspase-3 and downregulated p-JAK2, p-STAT3, and Bcl-2 levels. Berberine may induce MCF-7 cell apoptosis by regulating JAK2/STAT3 signaling in a concentration-dependent manner (Zhang et al., 2015b). 7β-(3-ethyl-*cis*-crotonoyloxy)-1α-(2-methylbutyryloxy)-3,14-dehydro-Z-notonipetranone, an ingredient isolated from *Tussilago farfara* L., was found to induce BC cell apoptosis by inhibiting JAK-STAT3 signaling and the expression of STAT3 target genes (Jang et al., 2019). In a study conducted by Ma et al. (2020), it was found that the mRNA expression of JAK2 and STAT3 was significantly decreased after curcumol treatment, suggesting that curcumol-induced apoptosis in BC cells might be realized through inhibition of the JAK2/STAT3 signaling pathway. In a study conducted in 2021, Feng et al. proposed that saikosaponin D can suppress cell growth and induce MCF-7 cell apoptosis, which may also be related to the JAK2/STAT3 pathway (Feng et al., 2021).

### Endoplasmic Reticulum Stress–Mediated Apoptosis

Hypoxia, starvation, Ca^2+^ imbalance, and other factors can induce ERS. During the ERS phase, PERK dissociates from GRP78/BiP and is activated by phosphorylation. PERK activation also leads to increased translation of transcription factors, such as ATF4, which further promotes the synthesis of the proapoptotic factor CHOP. CHOP downregulates the antiapoptotic protein Bcl-2 and promotes apoptosis. Moreover, CHOP promotes GADD45 expression, which triggers apoptosis by completely blocking protein synthesis. In 2014, Hou et al. (2014) proposed that fucoxanthin upregulated intracellular free calcium content and the activation of calpain in MCF-7 cells through ERS pathway, and treatment with fucoxanthin also promoted the activation of caspase-4/7/9 and release of CytoC, thereby inducing BC cell apoptosis. In 2020, Li et al. (2020) proposed that oleandrin can induce apoptosis of MCF-7 cells by downregulating Bcl-2 and upregulating Bax, Bim, and ERS-related proteins such as EIF2α, ATF4, and CHOP, suggesting that ERS plays an important role in this process. In 2018, Wu et al. (2018b) proposed that salviaflaside can induce MDA-MB-231 cell apoptosis by activating the ERS response to upregulate GRP78/CHOP and interfere with the Bcl-2/Bax balance.

### Other Pathway–Mediated Apoptosis

Multiple other signaling pathways can regulate BC cell apoptosis, such as the Wnt/β-catenin, Notch, and sirtuin-1 pathways. In 2012, curcumin, as the main ingredient of the spice turmeric isolated from the rhizomes of the plant *Curcuma longa* L., was reported to induce TNBC cell apoptosis by decreasing EGF receptor expression (Sun et al., 2012). In 2017, Zhao et al. (2017) also showed that curcumin suppressed cell growth and led to apoptosis in BT549 cells, and the mechanism was related to the inhibition of the Wnt/β-catenin signaling pathway. In 2014, Shao et al. (2014) reported that triptolide, a diterpene triepoxide compound, induced apoptosis *via* the Wnt/β-catenin signaling pathway. Ursolic acid is a natural pentacyclic triterpenoid compound. In 2012, Wang et al. (2012) demonstrated that ursolic acid inhibited FoxM1 expression and induced MCF-7 cell apoptosis, which was highly correlated with inactivation of cyclin D1/CDK4. In 2015, Fang et al. (2015) showed that calycosin promoted apoptosis of tumor cells MCF-7, possibly in part by reducing SIRT1 levels, thereby increasing p53 and cleaved caspase-3 expression to promote apoptosis. In 2015, Wei et al. (2015) reported that gambogic acid lysinate induced MCF-7 cell apoptosis in a dose-dependent manner by inhibiting SIRT1 protein expression levels and upregulating cleaved caspase-3 protein expression levels. In 2019, Cheng et al. (2019) indicated that icariin can significantly downregulate CDK2, CDK4, cyclin D1, and Bcl-2 and upregulate cleaved caspase-3 and PARP, thereby inducing apoptosis of the tamoxifen-resistant MCF-7/TAM BC cell line. In 2020, Yousuf et al. (2020) proposed that ellagic acid controlled cell proliferation and induced BC cell apoptosis by inhibiting CDK6. Moradi et al. (2020) also proposed in 2020 that flavonoid calycopterin can induce MDA-MB-231 cell apoptosis by increasing the caspase-3/8 expression. In addition, treatment with flavonoid calycopterin can also increase the ratio of Bax/Bcl-2.

Notch signaling can act as a therapeutic target to induce or resist cell apoptosis. When the Notch receptor is activated by DLL-1/2/3 and Jagged-1/2, the Notch intracellular domain (NIC) is released from the cell membrane. Then, the NIC translocates into the nucleus where it regulates the expression of target genes to induce a series of biochemical reactions. In 2021, Jiang et al. reported that quercetin could increase the expression of GAS5, Bax, and caspase-3 and decrease the expressions of Notch1, Jagged1, Hes1, and Bcl-2 in MCF-7 cells, suggesting that quercetin can induce BC cell apoptosis by the GAS5/Notch1 signaling pathway (Jiang et al., 2021). In 2019, a study by Lan et al. (2019) indicated that treatment with rhamnetin increased caspase-3/9 activity, upregulated p53 protein and miR-34a expression, and downregulated Notch-1 expression, suggesting that rhamnetin induced apoptosis of human BC cells through the miR-34a/Notch-1 signaling pathway. In 2021, Wang et al. (2021) reported that shikonin, a natural naphthoquinone isolated from *Arnebiae radix*, promoted the autoubiquitination and degradation of cIAP1 and cIAP2 to induce MDA-MB-231 cell apoptosis.

Abnormal expression of hedgehog ligands, overactivation of Smo proteins, and inappropriate disinhibition of Smo can lead to abnormal activation of the hedgehog pathway. Once the hedgehog pathway is activated, Gli transcription factors are transported into the nucleus in a full-length form and function as transcriptional activators to promote the abnormal expression of downstream VEGF, C-MYC, and other target genes, resulting in excessive cell proliferation and ultimately the occurrence of tumors. In 2020, Liu et al. (2020) showed that cordycepin induced apoptosis by inhibiting the hedgehog pathway, and this effect was associated with elevated levels of PUMA, CytoC, Fas, DR4/5, and cleaved caspase-3 and decreased levels of Bcl-2, XIAP, and PDGFR-α.

## Extracts From Natural Product–Induced Apoptosis in BC

In this study, we summarized the effects and mechanisms of extracts from natural products on the apoptosis of BC (Table 2). In 2010, Tandrasasmita et al. (2010) proposed that *Phaleria macrocarpa* (Scheff.) Boerl extract markedly decreased the PI3K/AKT signaling pathway and resulted in MDA-MB-231 cell apoptosis. In 2012, Vaz et al. (2012) reported that the methanol extract of *Suillus collinitus* (Fr.) Kuntze remarkably upregulated the levels of p53, p21 and cleaved PARP and downregulated the levels of Bcl-2 and XIAP in MCF-7 cell lines, suggesting that *S. collinitus*–induced apoptosis was realized by mediating p53. In a 2015 study conducted by Hosseini et al., the dichloromethane extract of *Scrophularia ningpoensis* Hemsl. *oxysepala* upregulated the mRNA expression of caspase-3 and caspase-9 in MCF-7 cells. Further, the expression study of caspase-9 mRNA confirmed that the fractions triggered apoptosis via the intrinsic mitochondrial pathway (Hosseini et al., 2015). In 2015, Zheng et al. (2015) proposed that ethyl acetate was isolated from the seeds of *Momordica cochinchinensis* (Lour.) Spreng. can induce cell apoptosis, which was correlated with high expression of p53, Bax, Bak, and Bad, along with downregulation of NF-κB. In a 2015 study conducted by Petchsak et al., *M. cochinchinensis* (Lour.) Spreng. aril extract induced BC cell apoptosis by increasing the expression of the proapoptotic gene Bax and enhancing caspase-6/8/9 activity (Petchsak and Sripanidkulchai, 2015). In 2015, Chen et al. (2015a) reported that extraction of isoflavones from chickpea *Cicer arietinum* L. sprouts induced apoptosis in SKBR3 and MCF-7 cells by increasing the expression of Bax, caspase-7/9, p53, and p21, and decreasing Bcl-2 expression. In 2016, Foo et al. reported that *Dillenia suffruticosa* (Griff.) Martelli root extract induced MDA-MB-231 cell apoptosis via activation of proapoptotic JNK1 and downregulation of antiapoptotic ERK1 and Bcl-2, increasing the Bax/Bcl-2 ratio and initiating the mitochondrial apoptosis pathway (Foo et al., 2016). In 2017, Liu et al. (2017b) reported that *Camellia sinensis* (L.) Kuntze polyphenols induced chromatin condensation, MMP reduction, ROS increase, and caspase-3/9 activation in BC cells, which indicated that *C. sinensis* (L.) Kuntze polyphenols induced mitochondrial pathway–mediated apoptosis. In 2017, Kello et al. (2017) found that peel polyphenol extracts induced apoptosis by increasing intracellular oxidative stress, activating p38 MAPK, and inhibiting ERK1/2 and AKT signaling pathways. In 2018, Zhang et al. (2018b) isolated three flavonoids from *Tephroseris kirilowii* (Turcz. ex DC.) Holub (isorhamnetin, genkwanin, and acacetin), which suppressed cell growth and induced apoptosis in MDA-MB-231 cells by downregulating the PI3K/AKT/mTOR/p70S6K/ULK signaling pathway. In 2018, Lin et al. (2018) proposed that the ethanol extract of *Antrodia cinnamomea* induced MCF-7 cell apoptosis by elevating the expression of miR-21-5p, miR-26-5p, and miR-30-5p and decreasing SKP2 mRNA expression. In 2018, Chun et al. (2018) found that the sesquiterpene lactones-enriched fraction of *Inula helenium* L. induced apoptosis *via* suppression of signal transducers and activators of the STAT3 signaling pathway in MDA-MB-231 cells. In 2019, Majumder et al. (2019) proposed that *Ricinus communis* L. fruit extract can contribute to apoptosis in MCF-7 and MDA-MB-231 cells by promoting the attenuation of antiapoptotic Bcl-2 and the accumulation of proapoptotic Bax and caspase-7 and PARP cleavage. In 2019, Lee et al. (2019) reported that *Fomes fomentarius* (L.Fr.) Kick. ethanol extracts induced apoptosis by inhibiting the PI3K/AKT pathway and activating the caspase pathway. In 2019, Kim et al. (2019) reported that the proapoptotic effect of *Toxicodendron vernicifluum* (Stokes) F.A. Barkley stokes extract in MCF-7 cells was attributed to p53, p21, and the intrinsic mitochondrial cascade. In a 2019 study, Chaudhry et al. (2019) reported that *Vitex agnus-castus* L. hexane and dichloromethane extracts induced T47D cell apoptosis by activating external and internal pathways, and this effect was concomitant with activation of caspase-8/9 and -3/7, upregulation of Bax and downregulation of Bcl-2. Khan et al. showed in a study in 2021 that *Phoenix dactylifera* L. ethanol extracts induced apoptosis in TNBC MDA-MB-231 cells. This effect was related to the upregulation of p53, Bax, and cleaved caspase-3 expression and downregulation of Bcl-2 and AKT/mTOR pathways (Khan et al., 2021).

**TABLE 2:** Extracts from natural products induced apoptosis in breast cancer.

Extracts	Compositions	Detail mechanisms		Cell model	Refs
		Upregulation	Downregulation		
*Phaleria macrocarpa* compound extract (DLBS1425) (5–50 µg/mL)	Phalerin	PTEN, p21, Bax, Bad, PUMA, activated caspase-9, cleaved PARP	p-Akt, BclxL	MDA-MB-231	Tandrasasmita et al. (2010)
*Suillus collinitus* methanolic extracts (25.2 ± 0.2 µg/mL)	Protocatechuic acid, p-hydroxybenzoic acid, cinnamic acid	p53, p21, cleaved PARP	Bcl-2, XIAP	MCF-7	Vaz et al. (2012)
*Scrophularia oxysepala* extracts (0–300 μg/mL)	Verbascosaponin, crokoelziside, isolated from these fractions	Caspase-3 mRNA, caspase-9 mRNA		MCF-7	Hosseini et al. (2015)
Ethyl acetate extracts of seeds of *Momordica cochinchinen* (ESMC2) (30, 60, 120 µg/mL)	Hexanoic acid, 2-heptenal, 2,4-nonadiena, 1,1′-carbonyldiimidazole, 1-oxaspiro [4,4]nonan-4-one, 5,5-dimethyl-cyclohex-3-en-1-ol, (E,E)-7,11,15, trimethyl-3-methylene-1,6,10,14-tetraene, l-(+), ascorbic acid 2,6-dihexadecanoate, 6-octadecenoic acid, octadecanoic acid, palmitin, octadecanoic acid 2,3-dihydroxypropyl ester	p53, Bax, Bak, Bad, cyclin B1, cyclin E, CDC2	NF-κB, Bcl-2, Mcl-1, p-ERK, p-p38, p-JNK, p-Akt	MDA-MB-231	Zheng et al. (2015)
*Momordica cochinchinensis* Aril extracts (50, 200, 400 µg/mL)	Lycopene, β-carotene	Bax, activated caspase-6, activated caspase-8, activated caspase-9		MCF-7	Petchsak and Sripanidkulchai, (2015)
Extracts from chickpea *Cicer arietinum* L. sprouts (20, 40, 60 µg/mL)	Ononin isoflavone, biochanin A-7-O-β-D-glucoside isoflavone, formononetin isoflavone, biochanin A isoflavone	Bax, caspase-7, caspase-9, p53, p21	Bcl-2 MCF-7	SKBr3	Chen et al. (2015a)
*Dillenia suffruticosa* dichloromethane root extracts (12.5, 25, 50 μg/mL)	Katonic acid, betulinic acid, koetjapic acid	JNK1	ERK1, Bcl-2	MDA-MB-231	Foo et al. (2016)
Green tea polyphenols (200, 300, 400 µg/mL)	Catechins	ROS, cleaved caspase-3, cleaved caspase-9		MCF-7	Liu et al. (2017b)
Fruit peel polyphenolic extracts flavine (F7) (150 µg/mL)	Myricetin, quercetin, kaempferol, rutin, isorhamnetine, catechin, epicatechin, malvidin-3-glucoside, caffeic acid, chrysin, galangin, apigenin, fisetin, luteolin, morin, anthocyanidins, stilbene resveratrol	Bax, cleaved PARP, activated caspase-7	Bcl-2	MCF-7	Kello et al. (2017)
Flavonoid compounds from *Tephroseris kirilowii* (IH:10–40 μM, GN:20–80µM, Aca:50–150 µM)	IH: isorhamnetin, GN: genkwanin, and Aca: acacetin	p53, cleaved caspase-3, LC3-II, ATG5	p-Cdc2, cyclin B1, Bcl-2, BclxL, PARP1, p62, PI3Kγ-p110, p-PI3K, p-Akt, p-mTOR, p-p70S6K, p-ULK	MDA-MB-231	Zhang et al. (2018b)
*Antrodia cinnamomea* extracts (100, 200 µg/mL)	Antcin K, antcin C, antcin B, methyl, antcinate B, eburi-coic acid, dehydroeburicoic acid	miR-21-5p, miR-26-5p, miR-30-5p	Skp2	MCF-7	Lin et al. (2018)
Hexane fraction from *Inula helenium* (HFIH) (8 μg/mL)	Alantolactone, isoalantolactone, igalan, dugesialactone, alloantolactone	p-JNK, activated caspase-3, cleaved PARP	Cyclin D1, CytoC, Bcl-2, p-ERK	MDA-MB-231	Chun et al. (2018)
*Ricinus communis* L. fruit extract (0.05, 0.1, 0.5, 1 µg/mL)	Ricinine, p-coumaric acid, epigallocatechin, ricinoleic acid	Bax, CytoC, caspase-7, cleaved PARP	Bcl-2	MDA-MB-231 MCF-7	Majumder et al. (2019)
*Fomes fomentarius* ethanol Extract Exerts (100 µg/mL)	Betulin	Cleaved caspase-9, cleaved caspase-3, cleaved PARP	MMP9, p-Akt, cyclin A/E, Bcl-2	MDA-MB-231	Lee et al. (2019)
Extracts of *Rhus verniciflua* Stokes (200, 300, 400 µg/mL)	Gallic acid	p53, p21, cleaved caspase-3, caspase-9, PARP, Bax	Bcl-2	MCF-7	Kim et al. (2019)
*Vitex rotundifolia* Fractions (10 µg/mL)	Vitexicarpin	Caspase-8, caspase-9, caspases-3/7, Bax	Bcl-2	T-47D	Chaudhry et al. (2019)
Extracts from Ajwa dates pulp (15, 20 mg/mL)	Maltose, catechin, myricetin, quercetin, β-sitosterol, digalacturonic acid, chlorogenic acid, β-carotene	p53, Bax, cleaved caspase-3	Bcl-2, p-Akt, p-mTOR	MDA-MB-231	Khan et al. (2021)

## Conclusion

Through a systematic summary, we found that numerous monomers/extracts have an anti-BC capacity, and the mechanism is closely associated with apoptosis. The mechanism of agent-induced apoptosis predominantly involves the mitochondrial pathway, PIK3K/AKT, NF-κB, MAPK, JAK-STAT3, and other pathways. Therefore, identifying potential anti-BC drugs from natural products is one of the most effective strategies. However, there are still some problems, and further studies should be performed in the future.

First, >50% of the studies mentioned in this review were performed *in vitro*. As we know, drugs undergo four processes *in vivo*, including absorption, distribution, metabolism, and excretion, all of which affect the therapeutic effect of drugs. Many drugs are effective *in vitro*. However, they have little or no effect *in vivo*. Therefore, animal experiments should be conducted to further confirm the proapoptotic effects of natural agents. Moreover, there is a lack of toxicity studies and clinical data on the majority of the reported natural products. Therefore, the therapeutic effects, side effects, and toxicity of these drugs should be the focus on the future studies. More time is required before these natural products are used clinically. Furthermore, the evidence regarding the mechanism of certain natural products mentioned in our article to promote BC cell apoptosis is unclear and inadequate. Further, we can use diversified detection methods, such as CRISPR-Cas9, single-cell sequencing, and proteomic technology, to clarify the mechanism in detail, which will be conducive to the development and use of these natural products. Finally, most natural products still have flaws, such as low bioavailability, poor solubility, and poor selectivity, which interfere with their clinical application (Zhang et al., 2021a). Therefore, special processes can be considered to overcome these deficiencies. We can modify the structure of the compound. For example, polar groups can be joined to increase the solubility. Nanoparticle drug delivery systems for natural products can increase bioavailability and reduce toxicity to other organs.

In conclusion, we reviewed current studies on natural products that induced BC cell apoptosis and summarized the pro-apoptotic mechanisms. We hope that our review can provide direction in the search for candidate drugs derived from natural products to treat BC by inhibiting cell apoptosis.
